# Hirudin suppresses hematogenous metastasis by targeting desmosome junction transition in circulating tumor cell clusters via HIF-1α–DSG2 signaling

**DOI:** 10.1038/s12276-025-01598-8

**Published:** 2025-12-12

**Authors:** Jueyao Zou, Junfeng Shi, Qiong Chen, Ziyan Zhu, Tongyao Hu, Zhengyu Zhang, Zhiqiang Pan, Fei Xu, Yong Zhu, Yuanyuan Wu, Yang Zhao, Aiyun Wang, Yin Lu, Yanhong Pan, Wenxing Chen

**Affiliations:** 1 Jiangsu Key Laboratory for Pharmacology and Safety Research of Chinese Materia Medica, School of Pharmacy, Nanjing University of Chinese Medicine, Nanjing, China; 2 Jiangsu Collaborative Innovation Center of Traditional Chinese Medicine Prevention and Treatment of Tumor, Nanjing, China; 3 Department of Oncology, Nanjing First Hospital of Nanjing Medical University, Nanjing, China; 4 School of Pharmacy, Nanjing University of Chinese Medicine, Nanjing, China; 5 National Medical Centre of Colorectal Disease, The Third Affiliated Hospital of Nanjing University of Chinese Medicine, Nanjing, China; 6 School of Medicine, Nanjing University of Chinese Medicine, Nanjing, China; 7 Jiangsu Joint International Research Laboratory of Chinese Medicine and Regenerative Medicine, Nanjing, China

**Keywords:** Breast cancer, Drug therapy

## Abstract

Circulating tumor cell (CTC) clusters, key in metastasis, rely on intercellular junctions for stability. However, the specific mechanisms governing intercellular connections within CTC clusters and the strategy targeting intercellular junctions to break CTC clusters remain elusive. Anticoagulants, commonly used to manage tumor-associated thrombosis, may potentially serve as CTC cluster dissociators, but their effects and mechanisms in inhibiting tumor metastasis are unclear. Hirudin, an anticoagulant peptide was used as a tool drug and found to inhibit breast tumor lung retention and colonization through its disruption of CTC clusters rather than directly inhibiting cell migration. Further research confirmed that within CTC clusters, desmosome junctions play a dominant role in maintaining CTC cluster formation with high expression of related proteins, while adhesion junctions express rarely. Desmoglein 2 (DSG2) mediates conversion between desmosome and adhesion junctions in CTC clusters. When DSG2 is highly expressed, the intercellular junctions within the CTC clusters are mainly composed of desmosomes. Reversely, low expression of DSG2 results in adhesion junctions. In addition, hypoxia-inducible factor-1 alpha (HIF-1α) positively controls DSG2-mediated desmosome junctions. Inhibiting HIF-1α promotes the conversion from desmosome to adhesion junctions, destabilizing CTC clusters. Hirudin inhibits hematogenous metastasis of breast cancer through suppression of HIF-1α-controlled DSG2-mediated desmosome junctions, ultimately leading to the disintegration of CTC clusters. Our findings highlight the therapeutic potential of targeting HIF-1α-controlled DSG2-mediated desmosome junction conversion and position hirudin as a promising CTC clusters dissociator optimized for the clinical prevention of breast cancer metastasis.

## Hirudin disrupts tumor cell clusters to prevent metastasis

Breast cancer is a leading cause of death among women, often due to the spread of cancer cells to other parts of the body. This study explores how hirudin, which prevents blood clots, might help stop this spread. The researchers focused on circulating tumor cell (CTC) clusters, which can lead to new tumors. The study involved experiments with breast cancer cells and mice to see how hirudin affects CTC clusters. The researchers found that hirudin can break up these clusters by interfering with proteins that help the cells stick together. This is important because breaking up the clusters could reduce the chance of cancer spreading. The results show that hirudin not only reduced the size and number of CTC clusters but also made it harder for them to form new tumors in the lungs.

This summary was initially drafted using artificial intelligence, then revised and fact-checked by the author.

## Introduction

Breast cancer is the most common cancer in women, with metastasis representing a predominant cause of mortality^1^. Owing to its complexity, current strategies for early diagnosis, recurrence prediction and therapeutic monitoring are still insufficient. Circulating tumor cells (CTCs) are common biomarkers for breast cancer diagnosis and prognostic evaluation, with their presence being responsible for early metastasis and relapse after adjuvant chemotherapy^2^. The invasion of tumor cells into the vasculature is an important way to achieve distant metastasis, which provides access to an avenue for CTCs to travel to the distal sites and colonize^3^. Compared to single cells, clustered CTCs, a metastatic mode formed by the aggregation of multiple CTCs, show higher metastatic efficiency and symbolize a worse prognosis^2^. This enhanced metastatic capacity is attributed to their greater invasiveness, as well as their superior ability to grow and survive within the circulatory system^4^. Cluster integrity during hematogenous metastasis is maintained through robust intercellular connections and elevated intercellular adhesion^5^. Dynamic modulation of junctional composition in response to environmental signals facilitates structural plasticity, enabling diverse types of collective cell movements, including cluster migration^6,7^. Furthermore, cellular junctions help cells coordinate their direction and speed^8^. The disruption of cell junction proteins reduces cluster formation and metastatic potential^2,9^. Therefore, agents that dissociate CTC clusters by disrupting junctional proteins may serve as valuable therapeutic tools to inhibit tumor metastasis. However, specific mechanisms governing intercellular junctions within CTC clusters remain elusive and the strategy targeting intercellular junctions to break CTC clusters is not well characterized.

Hypoxia is a crucial factor in solid tumor survival, promoting metastasis through epithelial–mesenchymal transition (EMT)^10^. Recent studies have revealed that EMT encompasses distinct intermediate states^11^. Among different hypoxic conditions, cyclic hypoxia is particularly potent as it increases hypoxia-inducible factor-1 alpha (HIF-1α) and tumor aggressiveness, fostering a partial EMT phenotype that enables collective cell migration as clustered CTCs or emboli^10,12^. The anoxic internal environment enhances cell adhesion and the formation of CTCs with high intravasation ability^13,14^. Notably, CTC aggregation exacerbates intratumoral hypoxia and acidosis^14^. This phenomenon may establish a mutually reinforcing relationship between CTC clusters and the hypoxic environment. HIF-1α not only promotes EMT but is highly upregulated in CTC clusters, with its suppression effectively inhibiting CTC cluster formation and subsequent pulmonary metastasis^15^. However, Chang et al.^16^ found that HIF-1α suppresses desmoglein 2 (DSG2), a component of desmosome-mediated adhesion, by recruiting EZH2 and SUZ12 to DSG2 promoters in hypoxic cells, increasing clustered CTCs and colonization. Moreover, DSG2 expression resumes when tumor cells exit hypoxia conditions^16^. These findings highlight the dynamic adjustment of intercellular junctions in response to different environments and stimuli. For instance, desmosomes and adhesion junctions act in concert to resist external mechanical forces, such as those caused by blood flow^17^. This raises questions regarding the role of HIF-1α in CTC clusters and whether regulating HIF-1α could inhibit their formation.

In clinical practice, anticoagulants are commonly utilized to prevent thrombosis in tumor patients. Notably, these agents may reduce the formation and retention of tumor emboli in the microvasculature to lower tumor metastatic potential^18^. Certain anticoagulant drugs, including aspirin, have been revealed to be useful for tumor prevention^19,20^. Moreover, they may be potential CTC cluster dissociators. Nierodzik et al.^21^ demonstrated that thrombin strengthens the adhesion of tumor cells to platelets, thereby promoting metastasis through the activation of platelet thrombin receptors and modulation of GPIIb–GPIIIa adhesion. Choi et al.^22^ discovered that thrombolytic agents such as urokinase can disrupt cell aggregates and decrease the incidence of CTC clusters. Among various anticoagulants, hirudin, an anticoagulant peptide derived from the medicinal leech (*Hirudo medicinalis*) and widely used in clinical practice, is recognized for its ability to inhibit cell adhesion without increasing bleeding risk^23–25^. This distinctive property renders hirudin a promising candidate for curbing tumor metastasis. Therefore, we chose hirudin for our study, with the expectation of identifying potential therapeutic agents from clinically available anticoagulants that could address breast cancer metastasis by interfering with CTC cluster formation within the vasculature.

In this study, we identified the anticoagulant peptide hirudin as a promising CTC cluster dissociator optimized for the clinical prevention of breast cancer metastasis. We further revealed that the HIF-1α–DSG2 axis mediates conversion between desmosome and adhesion junctions in CTC clusters, which is crucial for CTC cluster formation and disintegration during breast cancer metastasis. While elucidating the mechanism by which hirudin inhibits breast cancer metastasis, our findings indicate that HIF-1α and DSG2 may serve as potential targets for the development of CTC cluster dissociation agents.

## Materials and methods

### Cell lines and transfections

Breast tumor cell lines MCF-7, MDA-MB-231 and 4T1 were from the American Type Culture Collection and cultured in DMEM medium with 10% fetal bovine serum (FBS) and 1% penicillin–streptomycin at 37 °C and 5% CO₂. MCF-7-GFP cells, 4T1-luc cells and 4T1-Luc-GFP cells were achieved using lentiviral vectors (hU6-MCS-CBh-gcGFP-IRES-puromycin (LVCON313) and Ubi-MCS-SV40-firefly_Luciferase-IRES-Puromycin (LVCON101), Shanghai Genechem Co., Ltd).

For CTC cluster formation, cells were seeded at 1 × 10⁵ cells/ml in flasks and incubated on a shaker (50 rpm/min) to make the cells lose adhesion and spontaneously aggregate into cell clusters^26^. The culture conditions were adjusted to 5% oxygen and 2.5% FBS, with the addition of 10 ng/ml epidermal growth factor and 10 ng/ml basic fibroblast growth factor as growth factors^26^.

### Breast cancer in situ model

Female BALB/c mice (6–8 weeks old) from Shanghai Slaughter Laboratory Animal Co. were anesthetized with 2.5% isoflurane (RWD, R510-22-10), depilated near the fourth mammary gland and sterilized with ethanol. The 4T1-luc-GFP cells were resuspended in PBS and 5 × 10^5^ cells were injected into the right fourth mammary gland of mice. When the tumor volume approached 100 mm^3^, 17.5 U/10 g heparin, 5 mg/kg hirudin and 10 mg/kg hirudin were injected intraperitoneally once a day for 14 days. The peripheral blood was used for blood smear preparation to detect CTC clusters. The detailed procedure was as follows: mice were anesthetized and approximately 1 ml of peripheral blood was collected via cardiac puncture into EDTA-coated tubes. The blood sample was mixed with 3 ml of red blood cell lysis buffer and incubated on ice for 5 min. After centrifugation, the supernatant was discarded and the cell pellet was gently resuspended in 5 ml of PBS using a Pasteur pipette. The cell suspension was then transferred to a 35 mm culture dish and allowed to settle for 10 min. CTC clusters were identified under a fluorescence microscope as GFP-positive cellular aggregates comprising two or more cells with a diameter exceeding 20 μm. The primary tumors and lung tissues were subsequently harvested for RNA-sequencing (RNA-seq), histological analysis and micro-CT imaging.

### RNA-seq

Using Trizol reagent (Invitrogen), RNA was purified from primary tumor tissues and validated by an Agilent 2100 Bioanalyzer and agarose gel electrophoresis. The cDNA library was sequenced on the Illumina Novaseq6000 platform by Gene Denovo Biotechnology Co. Omicsmart (http://www.omicsmart.com) was applied for the bioinformatic analysis of the data.

### Transwell

To detect cell migration, a Transwell chamber (8 μm, 3422, Corning) was employed. In the upper chamber, 100 μl FBS-free DMEM medium containing 5 × 10^4^ cells were added. In the lower chamber, 600 μl DMEM medium with 20% FBS was included. The 4T1 cells and MCF-7 cells were treated with PBS and different concentrations of hirudin for 24 h. A cotton swab was used to remove nonmigrating cells in the upper chamber. After 15 min of fixing with 4% paraformaldehyde, the remaining cells were stained with 0.5% crystal violet for another 15 min. After washing, the membrane was excised from the Transwell chamber and mounted onto a glass slide using a mounting medium. The cells were visualized under a Zeiss microscope (Carl Zeiss AG).

### Wound healing assay

First, 5 × 10^5^ cells were seeded into a 6-well plate and incubated until they reached 85–95% confluence. A scratch was created using a 200 μl pipette tip, followed by two washes with PBS. FBS-free medium containing PBS or hirudin (25, 50 and 100 μg/ml) was added and the images were acquired at 0 h (t1) and 24 h (t2) using a Zeiss Axio vert A microscope. The scratch wound closure (%) was calculated as: (t1 − t2)/t1 × 100%.

### Tail vein pulmonary metastasis model

Female BALB/c mice aged 4–6 weeks, procured from Shanghai Slaughter Laboratory Animal Co. Ltd.,were utilized in this study. The 4T1-luc cells, 4T1-luc single cells, 4T1-luc cell clusters and hirudin pretreated 4T1-luc cell clusters (1.5 × 10^6^ cells) were injected into the tail vein to create a bioluminescent model of pulmonary metastasis. Mouse lung tissues were taken immediately after injection or 24 h later for Ki67 staining. On days 1, 2, 7 and 14, mice were respectively anesthetized and injected with 150 mg/kg d-fluorescein (CNSpharm, 115144-35-9) in the fundus venous plexus. Lung metastases were monitored by bioluminescence imaging using the IVIS spectroscopy system (Perkin Elmer). The body weights of the mice were recorded at 3-day intervals, and the animals were euthanized 2 weeks postmodeling. Lung tissues were collected and subjected to hematoxylin–eosin (H&E) staining, immunohistochemistry (IHC) and immunofluorescence.

### siRNA transfection

The HIF-1α siRNA and DSG2 siRNA were synthesized by Corues Biotechnology. MCF-7 cell clusters (1 × 10^5^ cells/well) were planted in a 12-well plate. The HIF-1α siRNA or DSG2 siRNA (50 nM) was diluted in Opti-MEM medium and mixed with lipofectamine 3000 reagent (Supplementary Table 1). After 6 h of transfection, the medium was refreshed with normal medium and incubated at 37 °C for 48 h. The CON siRNA served as a negative control.

### IHC

IHC was conducted utilizing an IHC Detection Kit (Supplementary Table 1). Paraffin-embedded tissue sections were subjected to deparaffinization and rehydration. Subsequently, antigen retrieval was carried out, followed by permeabilization and blocking procedures. Primary antibodies against Ki67, DSG2 and Plakoglobin (PG) (Supplementary Table 1) were incubated overnight at 4 °C. The biotinylated secondary antibody was incubated for 30 min and the antigen–antibody interaction was visualized using a streptavidin–biotin–HRP system with diaminobenzidine as the substrate. Nuclei were counterstained with hematoxylin. Images were obtained with an Aperio Scanner System (Leica) at a magnification of 20×.

### Immunofluorescence

The cell clusters were adhered to adhesive slides by centrifugation at 500*g* for 3 min using a cell smear centrifuge (Kaida, TD4B), followed by fixation in 4% paraformaldehyde and permeabilized with 0.1% Triton X-100. Following a 1-h block with 1% BSA, the samples were subjected to overnight incubation with primary antibodies at 4 °C. The primary antibodies used were as follows: PG, zona occludens 1(ZO-1), DSG2, carcinoembryonic antigen related cell adhesion molecule 6 (CEACAM6), vimentin, programmed cell death-ligand 1 (PD-L1), HIF-1α, E-cadherin and β-catenin (Supplementary Table 1). Following PBS washes, the samples were incubated with appropriate fluorescent secondary antibodies for 2 h at room temperature. The secondary antibodies employed included goat anti-mouse IgG H&L (FITC) and goat anti-rabbit IgG H&L (TRITC) (Supplementary Table 1). For lung tissues, the paraffin-embedded sections were used for the immunofluorescence staining after antigen retrieval. DAPI was applied to indicate the cell nucleus. Images were acquired using a Zeiss Axio vert A1 microscope.

### Transmission electron microscopy

Tumor cell clusters were rinsed with DPBS, followed by fixation in 2.5% glutaraldehyde at 4 °C for a minimum of 16 h. Samples were subsequently forwarded to the Research Dog Instrument Testing Platform for visualization using a JEM1400 transmission electron microscope operated at 120 kV.

### WB

For western blotting (WB), cells were disrupted using RIPA lysis buffer supplemented with protease and phosphatase inhibitors. A BCA assay kit was applied to determine the protein concentration. Proteins were then separated by SDS–PAGE. After transferring the proteins onto a PVDF membrane, the membrane was blocked with 5% skim milk and then incubated with the specified primary antibodies: PG, PD-L1, β-catenin, CEACAM6, DSG2, vimentin, HIF-1α and GAPDH. Goat anti-rabbit IgG (H&L)-HRP and goat anti-mouse IgG (H&L)-HRP secondary antibodies were employed (Supplementary Table 1). The blots were visualized with a Gel Imaging System (Bio-Rad, ChemiDoc XRS+), followed by quantification of protein expression levels.

### Limiting dilution assay

MCF-7 cell clusters were cultured for 24 h, either with or without hirudin treatment. To ensure uniform cell numbers, the clusters were dissociated into single cells using trypsin and then subjected to serial dilution. The cells were subsequently seeded at a density of 2000 cells/well in 6-well plates and cultured for 7 days. Colonies, defined as cellular aggregates containing more than 50 cells, were quantified using a Leica stereo fluorescence microscope.

### EdU incorporation assay

A BeyoClick EdU Cell Proliferation kit (Supplementary Table 1) with Alexa Fluor 555 was used. Briefly, MCF-7 cell clusters were cultured with or without hirudin for 24 h. Subsequently, cell clusters were treated with EdU for 2 h, fixed with 4% paraformaldehyde for 15 min and permeated with 0.3% Triton X-100 for another 15 min. After a 30-min incubation of the click reaction mixture at room temperature from light, the cell nucleus was stained with DAPI for 10 min.

### Cell adhesion assay

After 24 h incubation of MCF-7 adherent cells (8 × 10^4^) in the 96-well plates, the dissociated MCF-7-GFP cell clusters (2 × 10^4^) were added and incubated for another 30 min. Nonadherent cells were then removed by washing with PBS. Adhesion efficiency was assessed by counting the adherent GFP-labeled cells under a fluorescent microscope and analyzing the images with ImageJ software. Five fields of view were analyzed per group.

### Molecular docking

Protein–protein docking was conducted using the HDOCK server (http://hdock.phys.hust.edu.cn/). The process included an initial sampling of binding modes via a fast Fourier transform (FFT)-based global search, followed by evaluation using an improved iterative knowledge-based scoring function^27^. For the protein-protein docking, the X-ray crystal structures of HIF-1α (PDB ID: 3KCX) served as the receptor, while hirudin (PDB ID: 4MLF) was used as the ligand. These structures were obtained from the protein data bank database (https://www.rcsb.org/). Discovery Studio software was used to visualize the docking results of the members with the highest scores.

### MD simulation

Molecular dynamics (MD) calculations were performed using GROMACS 2018 software. The TIP3P water model was used as the solvent for the complex, with a 7-Å-thick layer of water molecules added around the solute space. An appropriate amount of sodium ions was added to the system to balance the excess negative charge, ensuring the electroneutrality of the system. The CHARMM force field was applied to the simulation. Before the MD simulation, the system energy was optimized using the steepest descent method to eliminate large energy collisions between molecules. Then, the energy was minimized a second time using the conjugate gradient method. The system was heated from 50 K to 300 K and pre-equilibrated for 50 ps at constant temperature and pressure.

A 1 ns MD simulation was performed to bring the entire system to an equilibrium state. The complexes were then simulated for 30 ns using periodic boundary conditions. The particle mesh Ewald method was used to handle long-range electrostatic interactions, with the cutoff radius for nonbonded interactions set to 14 Å, the timestep set to 0.5 fs and the coordinate trajectories recorded every 10 ps. The trajectory processing was conducted using VMD 1.9.4.

### CETSA

For the cellular thermal shift assay (CETSA), the lysate of MCF-7 cell clusters was collected and centrifuged at 20,000 *g*/min, 4 °C for 20 min to obtain the supernatant. The supernatant was equally divided and treated with PBS or 5000 μg/ml hirudin for 30 min at room temperature. The supernatant was then equally divided into 12 parts and heated under 42 °C, 44 °C, 46 °C, 48 °C, 50 °C, 52 °C, 54 °C, 56 °C, 58 °C, 60 °C, 62 °C and 64 °C for 3 min, respectively. After cooling at room temperature for 3 min, new supernatants were obtained by centrifuging at 20,000*g*/min, 4 °C for 20 min to detect the protein level of HIF-1α by WB.

### DARTS assay

The drug affinity responsive target stability (DARTS) assay was conducted following a published protocol^28^. MCF-7 cell clusters were collected and lysed with M-PER Mammalian Protein Extraction Reagent (Supplementary Table 1). The lysate was centrifugated at 18,000 *g* for 20 min at 4 °C. After centrifugation at 18,000 *g* for 20 min at 4 °C, the supernatant was split equally and incubated in the presence or absence of 5000 μg/ml hirudin at room temperature for 30 min. The supernatant, with or without hirudin, was divided equally into six portions, to which varying concentrations of pronase were added and incubated at room temperature for 30 min. The reaction was stopped by adding 0.5 M EDTA on ice for 15 min. After adding the loading buffer, the samples were heated at 70 °C for 10 min for the WB experiment.

### Statistical analysis

All experiments were conducted a minimum of three times, with results expressed as mean ± s.d. GraphPad Prism 6 software was applied for the statistical analyses. For pairwise comparisons, parametric data were analyzed using a two-sample *t*-test. For comparisons involving multiple groups, a one-way ANOVA was applied, followed by the Bonferroni correction for post hoc analysis. *P* < 0.05 was considered statistically significant (*P < 0.05, **P < 0.01 and ***P < 0.001).

## Results

### Result 1. CTC clusters are potential targets for hirudin to inhibit spontaneous lung metastasis of breast tumor cells

**Fig. 1:**
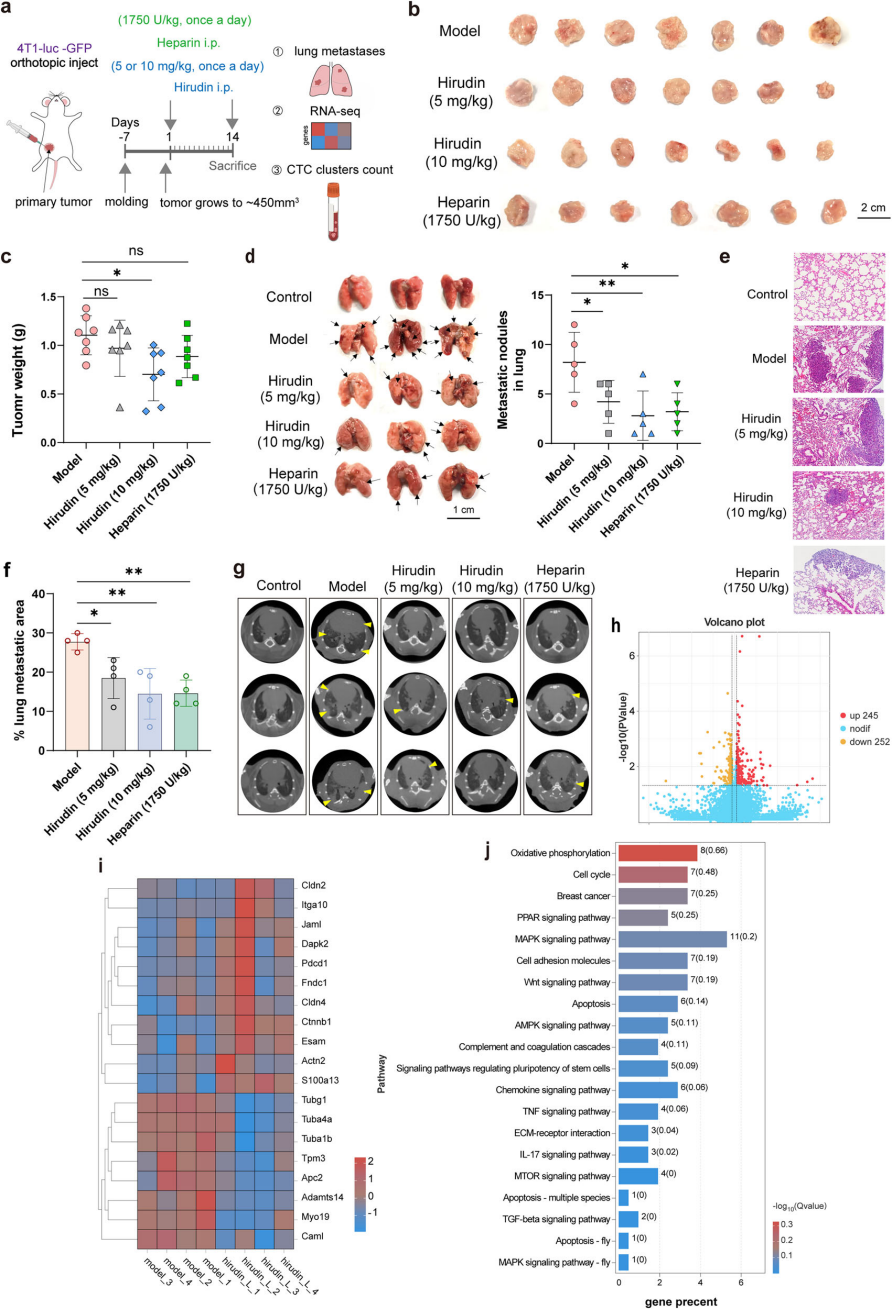

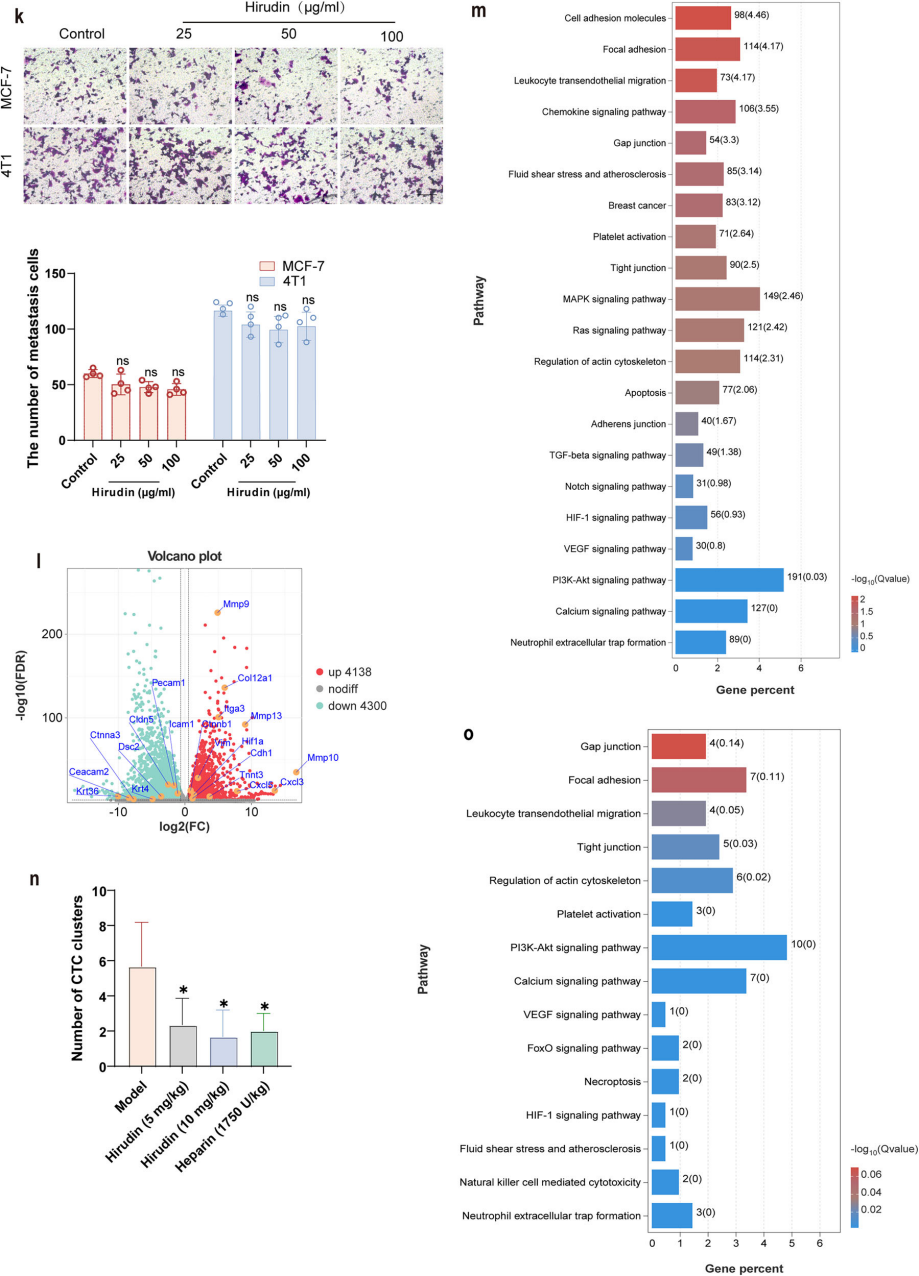
CTC clusters are potential targets for hirudin to inhibit spontaneous lung metastasis of breast tumor cells. **a** The flow chart of animal experiments. **b**,**c** Tumor size (**b**) and tumor weight (**c**) in an orthotopic tumor model mice established with 4T1-GFP-luc cells after hirudin or heparin administration. *n* = 7. **d** Representative lung tissue images and metastatic nodule counts after killing. *n* = 5. **e**–**g** H&E staining (**e**), quantification (**f**) and micro-CT (**g**) analysis of lung metastases in a 4T1-GFP-luc tumor model after hirudin or heparin treatment. **h** A volcano plot of differentially expressed genes in the primary tumor tissues from the model and the 5 mg/kg hirudin groups. **i** A heat map of metastasis-related genes in primary tumor tissues from model and 5 mg/kg hirudin groups. **j** KEGG enrichment analysis of differentially expressed genes in primary tumor tissues from model and 5 mg/kg hirudin groups. *n* = 4. **k** The migration of MCF-7 and 4T1 cells treated with hirudin for 24 h was determined by the transwell assay. *n* = 4. **l** A volcano plot of differentially expressed genes between para-cancer and tumor tissue in model mice. *n* = 4. **m** KEGG enrichment analysis showing CTC cluster formation-related pathways based on differentially expressed genes between para-cancer and tumor tissue. **n** The number of CTC clusters in 1 ml of peripheral blood was determined from orthotopic tumor models established using 4T1-GFP-luc cells. *n* = 3. **o** KEGG enrichment analysis conducted on differentially expressed genes in primary tumor tissues from the model group and 5 mg/kg hirudin group identified pathways related to the formation of CTC clusters. *n* = 4. Data are mean ± s.d., *P < 0.05, **P < 0.01 versus model group. i.p., intraperitoneal; ns, not significant.

To confirm whether anticoagulants have antitumor metastatic effects, we selected the more clinically used heparin and the clinically safer anticoagulant peptide hirudin and observed their effects on spontaneous lung metastasis of 4T1-luc-GFP cells (Fig. 1a). While 10 mg/kg hirudin significantly inhibited both primary tumor growth and metastasis, the lower 5 mg/kg hirudin dose and 1,750 U/kg heparin selectively suppressed pulmonary metastatic foci and nodules without affecting primary tumor growth (Fig. 1b–g and Supplementary Fig. 1a). This observation piqued our curiosity regarding the mechanisms by which 5 mg/kg hirudin and 1,750 U/kg heparin exert their inhibitory effects on breast tumor metastasis. RNA-seq showed that 5 mg/kg hirudin significantly upregulated 245 genes and notably downregulated 252 genes compared to the model group (Fig. 1h). These differentially expressed genes were involved in processes critical to tumor metastasis such as cell adhesion, cell migration and blood vessel development (Fig. 1i,j and Supplementary Fig. 1b). Similarly, heparin altered the expression of genes related to cell migration, vascular development and extracellular matrix integrity (Supplementary Fig. 1c–f). Strikingly, at concentrations not exceeding 100 µg/ml (where hirudin did not affect the proliferation of breast tumor cells), hirudin failed to inhibit the migration of breast tumor cells in either the horizontal or vertical direction (Fig. 1k and Supplementary Fig. 1g–i). Given that tumor cells can exist in the circulation both as individual cells and as more metastatic CTC clusters, we analyzed cluster-associated pathways. RNA-seq of para-cancerous and tumor tissues from model mice displayed significant alterations in cell adhesion and aggregation genes involved in CTC cluster formation (Fig. 1l,m and Supplementary Fig. 1j,k), suggesting a cluster-targeted mechanism. To verify our suspicions, we detected CTC clusters and found that both 5 mg/kg hirudin and 1,750 U/kg heparin significantly reduced the number of CTC clusters in the peripheral blood of model mice (Fig. 1n). Importantly, genes that were significantly different in primary tumor tissues after 5 mg/kg hirudin treatment compared to those in model mice were involved in pathways and biological processes such as cell junctions and cell adhesion, which are crucial for CTC cluster formation (Fig. 1o and Supplementary Fig. 1l). Similar findings were observed in the heparin group (Supplementary Fig. 1m,n). Considering these findings, we hypothesize that anticoagulants have the potential to inhibit tumor metastasis and that CTC clusters mediate the antimetastatic effects of the anticoagulant peptide hirudin on breast tumor cells.

### Result 2. Hirudin suppresses tumor retention and colonization by disrupting CTC clusters

**Fig. 2:**
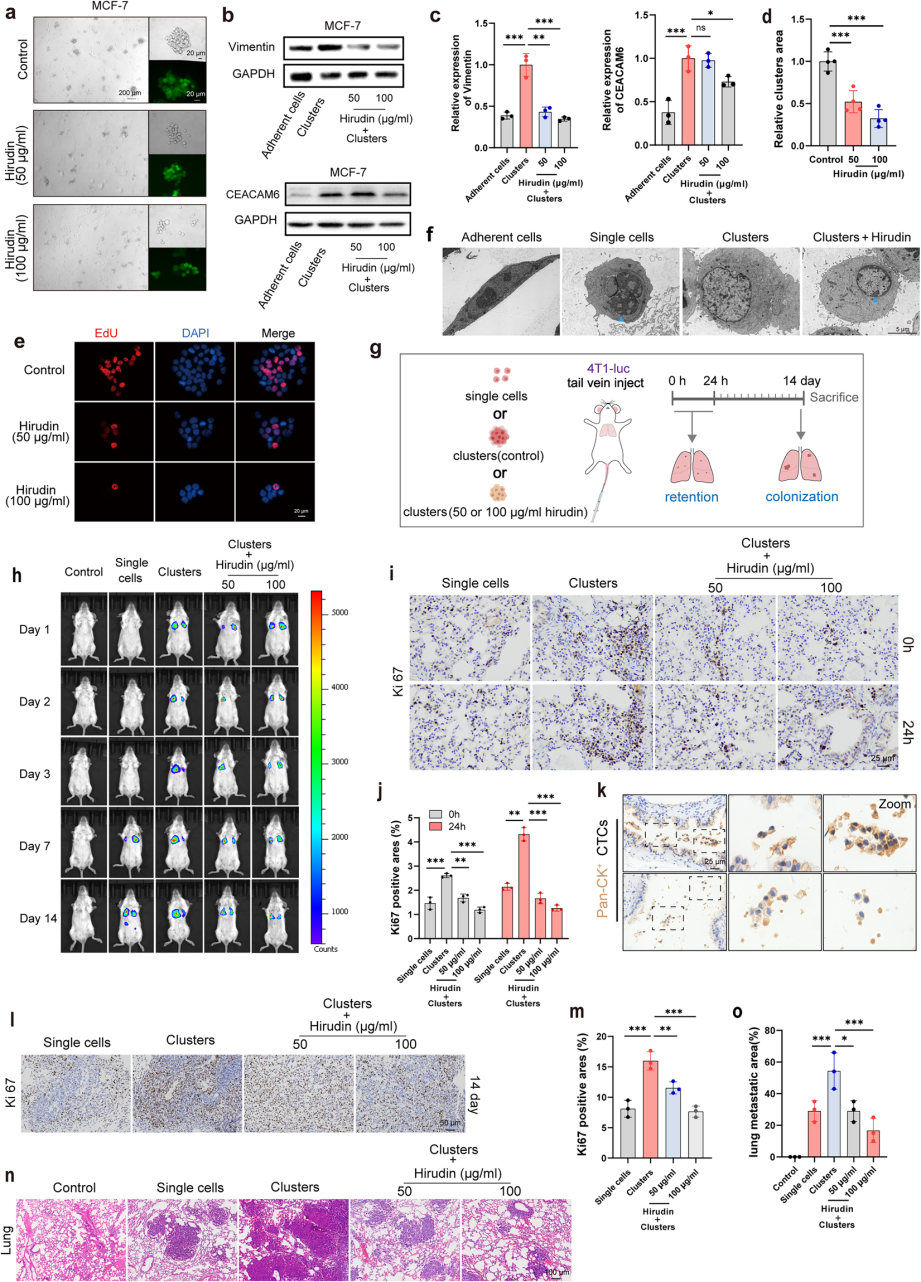
Hirudin suppresses tumor retention and colonization by disrupting the CTC clusters. **a** MCF-7-GFP cells were used to create in vitro CTC clusters. After treating with 50 μg/ml or 100 μg/ml hirudin for 24 h, the formation of CTC clusters was observed. *n* = 3. **b** The protein expression of vimentin and CEACAM6 after 24-h treatment of hirudin or PBS. *n* = 3. **c** Quantitative analysis of vimentin and CEACAM6 protein expression. **d** Quantitative analysis of the relative cluster area of MCF-7 cell clusters. *n* = 4. **e** CTC clusters were blown apart and the proliferation of tumor cells within the clusters was examined by Edu staining. *n* = 3. **f** Transmission electron micrographs displaying the morphological characteristics of tumor cells in different states. The arrow indicates cells in apoptosis or premature apoptosis. **g** A schematic illustrating the establishment of the lung metastasis model via tail vein injection of 4T1-luc single cells or cell clusters pre-treated with hirudin or PBS for 24 h. **h** Representative bioluminescence images (IVIS spectral system) monitoring lung metastases on days 1, 2, 3, 7 and 14 after injection. *n* = 7. **i**–**k** Ki67 (**i, j**) and pan-CK (**k**) immunohistochemical images of lung tissue in retention model, and quantitative analysis of Ki67 (**j**). *n* = 3. **l**, **m** Ki67 immunohistochemical images (**l**) and quantitative analysis (**m**) of lung tissue in the colonization model. *n* = 3. **n**,**o** Representative HE staining (**n**) and quantitative analysis (**o**) of lung metastases in colonization model mice. *n* = 3. Data are mean ± s.d., *P < 0.05, **P < 0.01, ***P < 0.001 versus clusters.

To further investigate whether hirudin inhibits tumor metastasis by targeting CTC clusters, we constructed in vitro CTC clusters that closely mimicked the morphology of peripheral blood CTC clusters from tumor-bearing mice (Supplementary Fig. 2a,b). In the bloodstream, CTC clusters are subjected to various challenges, including blood shear forces, anoikis and immune cell attacks. To survive these threats, CTC clusters often upregulate key biomarkers related to these survival mechanisms^26^. Our results confirmed that hirudin not only significantly reduced the size and invasion ability of CTC clusters but also downregulated the expression of proteins associated with anti-anoikis, immune escape and EMT, namely CEACAM6, PD-L1 and Vimentin (Fig. 2a–dand Supplementary Fig. 2c–e). Successful metastasis requires not only survival in circulation but also effective retention and colonization at distant sites. In vitro experiments revealed that 50 μg/ml and 100 μg/ml hirudin successfully reduced the proliferation capacity of tumor cells within CTC clusters without affecting the cell viability of adherent tumor cells under normal, noncluster-forming conditions (Fig. 2eand Supplementary Figs. 1gand 2f–j). Interestingly, when tumor cells lost their ability to adhere, single suspended cells exhibited apoptotic features (Fig. 2fand Supplementary Fig. 2k,l). However, these apoptotic features disappeared when cells formed clusters (Fig. 2fand Supplementary Fig. 2k,l). The use of hirudin was able to induce premature apoptotic features such as nuclear crumpling and chromatin border aggregation in clustered cells (Fig. 2f and Supplementary Fig. 2k,l). In the tumor retention model, both bioluminescence imaging and immunohistochemical staining for Ki67, PD-L1 and vimentin showed that CTC clusters were more efficiently retained in lung tissues than single cells (Fig. 2g–k and Supplementary Fig. 2m–o). Additionally, during metastasis, CTC clusters appeared to maintain their aggregate structure and become entrapped within the pulmonary tissue (Fig. 2k). However, these effects were able to be reversed by hirudin (Fig. 2g–k and Supplementary Fig. 2m–o). Compared to untreated CTC clusters, the colonization ability of hirudin-treated CTC clusters in the lung tissues was significantly weakened, and the effect was not temporary (Fig. 2g,h,l,mand Supplementary Fig. 2p–s). H&E staining further confirmed that lung metastasis was significantly reduced in hirudin-treated CTC clusters (Fig. 2n,o). Collectively, these results indicated that hirudin achieved the effect of inhibiting tumor metastasis by disrupting CTC clusters and reducing their retention and colonization at metastatic sites.

### Result 3. Hirudin inhibits the formation of CTC clusters by impeding adhesion between breast tumor cells

**Fig. 3:**
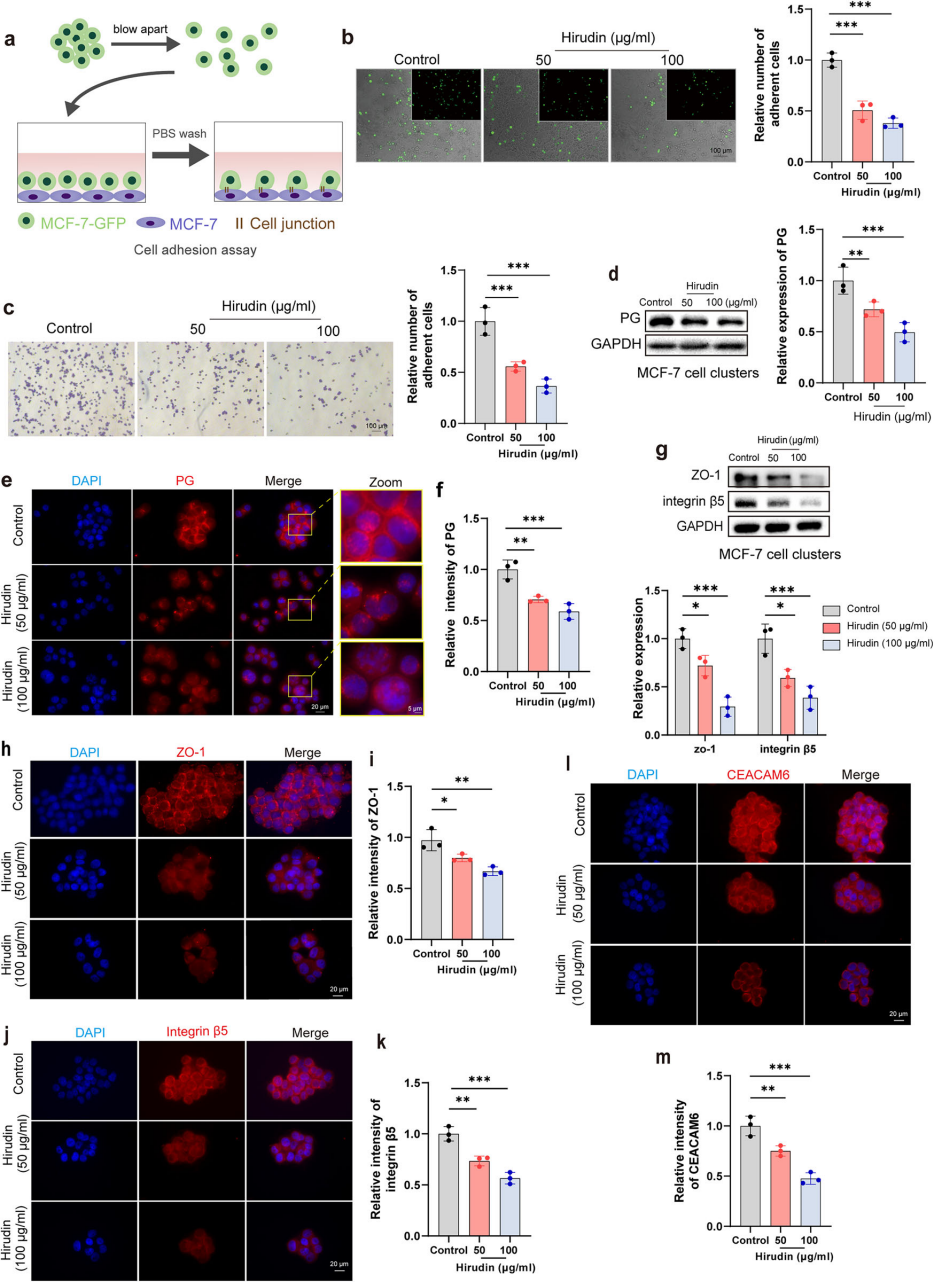
Hirudin inhibits the formation of CTC clusters by impeding adhesion between breast tumor cells. **a** Schematic diagram of the cell-cell adhesion assay. Upper layer: MCF-7-GFP; lower layer: MCF-7. **b** A cell–cell adhesion assay was performed to evaluate the intercellular adhesion in MCF-7 clusters following 24 h of hirudin treatment. Representative fluorescence images were taken, and adherent MCF-7-GFP cells were quantified. *n* = 3. **c** A cell–fibronectin adhesion assay was used to assess the extracellular matrix attachment capacity of cells within MCF-7 clusters following 24 h hirudin treatment. *n* = 3. **d** WB analysis of PG expression in 24 h hirudin-treated MCF-7 cell clusters with grayscale values normalized to GAPDH. *n* = 3. **e**,**f** Immunofluorescence staining of PG in 24 h hirudin-treated MCF-7 cell clusters (**e**) with mean fluorescence intensity quantification (**f**). Red, PG; blue, nucleus. *n* = 3. **g** WB analysis of ZO-1 and integrin-β5 expression in hirudin-treated MCF-7 cell clusters. *n* = 3. **h**–**m** Immunofluorescence staining (**h**, **j** and **l**) and mean fluorescence intensity quantification (**i**, **k** and **m**) of ZO-1 (**h** and **i**), integrin-β5 (**j** and **k**) and CEACAM6 (**l** and **m**) in 24 h hirudin-treated MCF-7 cell clusters. Red, ZO-1/integrin-β5/CEACAM6; blue, nucleus. *n* = 3. Data are mean ± s.d., *P < 0.05, **P < 0.01, ***P < 0.001 versus control.

Cell adhesion is fundamental for cell aggregation, and robust intercellular adhesion is essential for the maintenance of tumor cell clusters in circulation. Building on our RNA-seq data, we were eager to explore whether hirudin could inhibit the formation of CTC clusters by modulating adhesive properties. We observed weaker cluster adhesion in the hirudin-treated group, which was consistent in the fibronectin adhesion assay (Fig. 3a–c). Accumulating evidence implies that PG is upregulated in CTC clusters compared to single CTCs and plays a key role in intercellular connections^29,30^. We found that hirudin treatment led to a marked reduction in PG protein expression, particularly at the junctions of MCF-7 and 4T1 cell clusters (Fig. 3d–fand Supplementary Fig. 3a–c). This reduction in PG expression was accompanied by a significant decrease in the expression of other cell adhesion proteins such as ZO-1, integrin β5 and CEACAM6 (Fig. 3d–m and Supplementary Fig. 3d). Notably, 100 μg/ml hirudin effectively dispersed CTC clusters by strongly inhibiting the expression of these cell adhesion proteins (Fig. 3g–mand Supplementary Fig. 3d). These findings suggested that the disruption of intercellular adhesion is a critical process by which hirudin interferes with CTC cluster formation.

### Result 4. CTC cluster formation and separation are modulated by DSG2-mediated desmosome–adhesion junction conversion

**Fig. 4:**
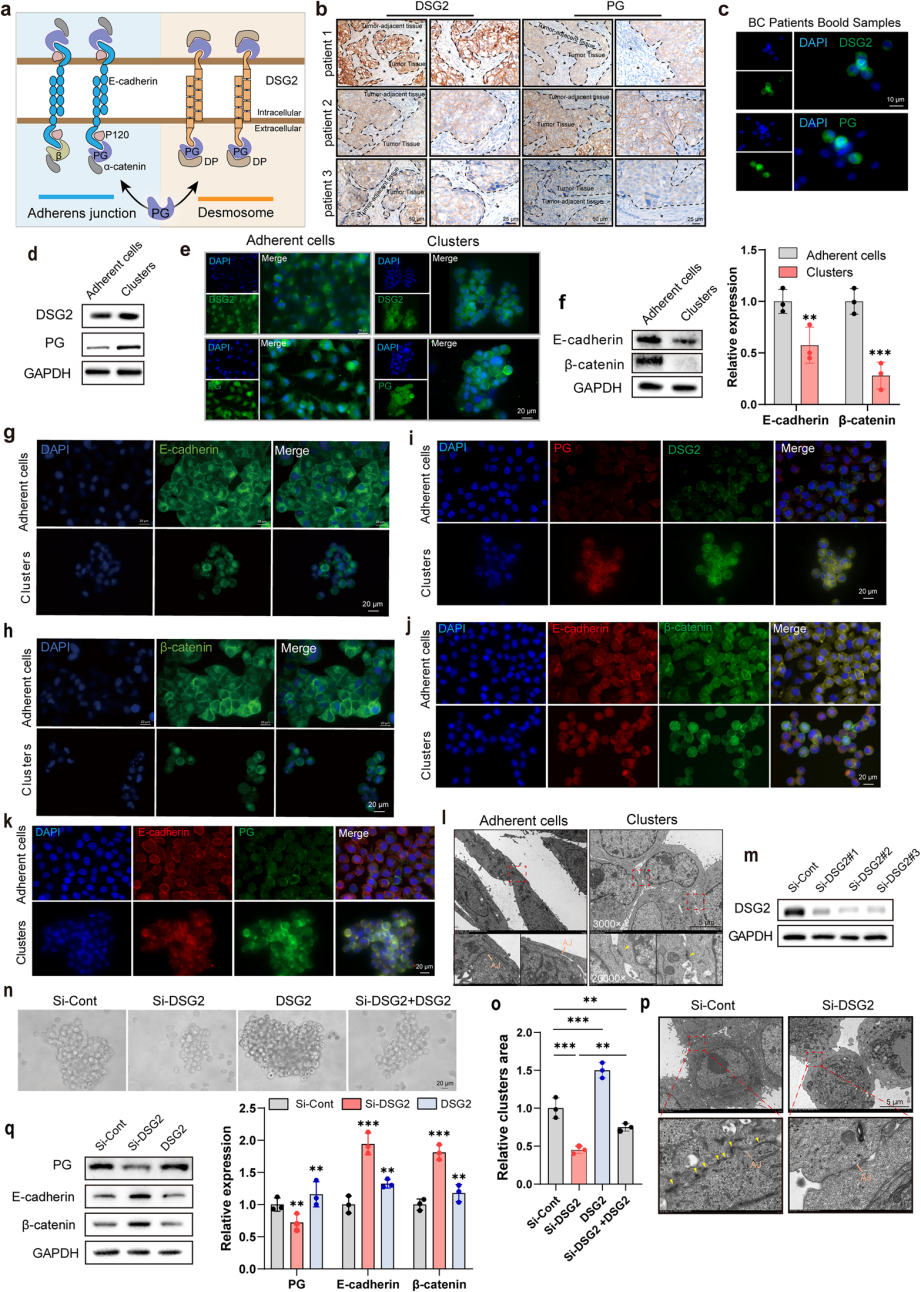
CTC cluster formation and separation are modulated by DSG2-mediated desmosome–adhesion junction conversion. **a** A schematic diagram of desmosomes and adherens junctions. **b**,**c** The expression of DSG2 and PG proteins in primary tumors (**b**) and blood samples (**c**) from patients with breast cancer. **d** WB analysis of DSG2 and PG expression in MCF-7 adherent cells and cell clusters. *n* = 3. **e** Immunofluorescence analysis of DSG2 and PG expression in MCF-7 adherent cells and cell clusters. *n* = 3. **f** WB analysis of the expression differences of E-cadherin and β-catenin in MCF-7 adherent cells and cell clusters, with grayscale quantification. *n* = 3. **g**,**h** Immunofluorescence analysis of E-cadherin (**g**) and β-catenin (**h**) expression in MCF-7 adherent cells and cell clusters, with quantification of mean fluorescence intensity. *n* = 3. **i**–**k** Immunofluorescence analysis showing the expression and colocalization of DSG2 and PG (**i**), E-cadherin and β-catenin (**j**) and E-cadherin and PG (**k**) in MCF-7 adherent cells and cell clusters. *n* = 3. **l** Transmission electron microscopy images of desmosomes (yellow arrows) and adherent junctions (AJ) in MCF-7 adherent cells and cell clusters. *n* = 3. **m** WB analysis of DSG2 expression. *n* = 3. **n**,**o** The formation of CTC clusters following DSG2 knockdown or recombinant DSG2 protein treatment. Representative bright‑field images of CTC clusters (**n**), and quantitative analysis of cluster area (**o**). *n* = 3. **p** Transmission electron microscopy showing the desmosomes (yellow arrows) and AJ in MCF-7 cell clusters with or without DSG2 knockdown. *n* = 3. **q** WB analysis of PG, E-cadherin and β-catenin expression after DSG2 knockdown or recombinant DSG2 treatment, with grayscale quantification. *n* = 3. Data are mean ± s.d., **P < 0.01, ***P < 0.001 versus Si-DSG2.

In addition to adhesion, the diverse intercellular junctions also contribute to cell aggregation and are crucial for maintaining the integrity and migration of cellular clusters within the vasculature. Unfortunately, the specific cellular junctions involved in CTC cluster formation remain unclear. PG, a common junctional plaque protein, is present in a strong junction called desmosome and functionally stabilizes DSG2, a partner of this junction^31^ (Fig. 4a). In addition, PG can substitute for β-catenin in E-cadherin–catenin adhesion complexes, which are involved in another intercellular junction called the adhesion junction^32^ (Fig. 4a). In our study, PG expression was inhibited by hirudin in MCF-7 and 4T1 cell clusters (Fig. 3d–f and Supplementary Fig. 3a,b). Combining these pieces of information, we first investigated the role of desmosome and adhesion junctions in CTC clusters. In a cohort of ten patients with breast cancer with distant metastasis, we observed higher expression of DSG2 and PG in tumor tissues compared to adjacent normal tissues (Fig. 4b). Importantly, blood samples from two patients revealed elevated DSG2 and PG in cell clusters versus single cells (Fig. 4c). As anticipated, DSG2 expression was markedly higher in PG-enriched breast tumor cell clusters compared to adherent cells (Fig. 4d,eand Supplementary Fig. 4a–c). However, the expression levels of E-cadherin and β-catenin were significantly downregulated in clusters compared to adherent cells (Fig. 4f–h and Supplementary Fig. 4d–i). Further experiments revealed that cell clustering did not significantly enhance the colocalization of PG and DSG2, but significantly weakened the colocalization of E-cadherin and β-catenin (Fig. 4I,jand Supplementary Fig. 4j,k). Although CTC clusters have stronger membrane colocalization of E-cadherin with PG than adherent cells, E-cadherin expression was significantly decreased in CTC clusters (Fig. 4f,g,k and Supplementary Fig. 4d–h). Therefore, the formation of CTC clusters appeared dominated by robust desmosome junctions rather than fragile adhesive junctions.

To further validate our speculation, we utilized transmission electron microscopy to observe intercellular junctions at the ultrastructural level. Compared to adherent cells, cells within the CTC cluster showed obvious desmosome junctions (Fig. 4l). After successfully knocking down desmosome component DSG2 in MCF-7 cells using siRNA, we found that the cell clustering and adhesion abilities were significantly decreased (Figs. 4m–oand 5aand Supplementary Fig. 4l–n). Moreover, DSG2 knockdown resulted in an obvious reduction in cellular junctions, particularly desmosome junctions, with a slight increase in adhesion junctions (Fig. 4p). The addition of the recombinant DSG2 protein reversed the attenuation of cell clustering caused by DSG2 knockdown (Fig. 4n,o). To further illustrate the regulatory role of DSG2 between the two junction types, we measured the expression of E-cadherin, β-catenin and PG in DSG2 knockdown cells. The knockdown of DSG2 was followed by upregulation of E-cadherin and β-catenin and downregulation of PG in CTC clusters (Fig. 4q). The phenomenon was reversed by adding recombinant DSG2 protein (Fig. 4q). Overall, our data indicated that DSG2 orchestrates the formation and disassembly of CTC clusters by mediating the switch between desmosome and adhesion junctions.

**Fig. 5:**
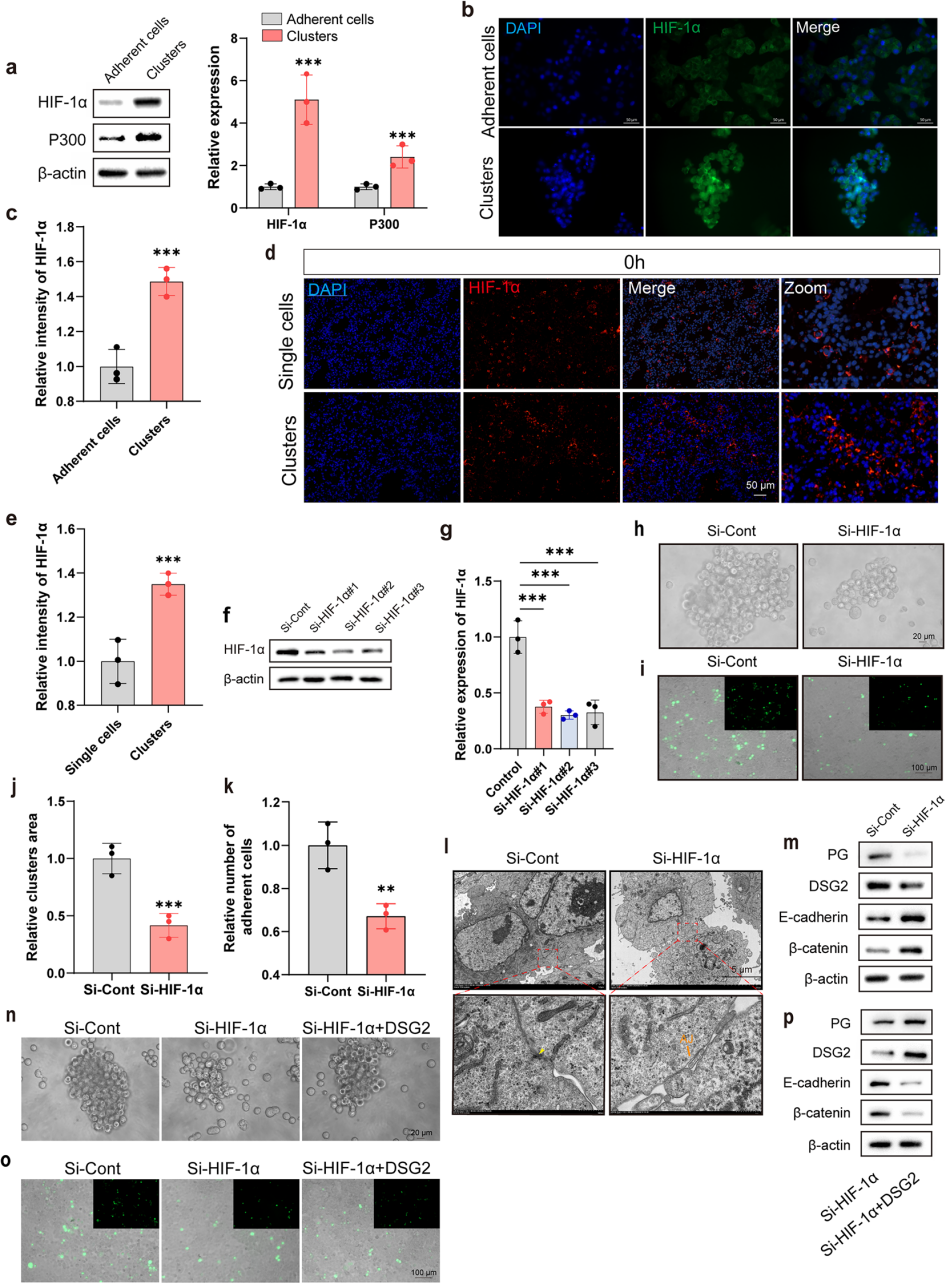
HIF-1α drives the transition from adhesion junction to desmosome junction through DSG2 to aggravate CTC clustering. **a** WB analysis of HIF-1α and P300 expression in MCF-7 adherent cells and cell clusters with grayscale quantification. *n* = 3. **b**,**c** Immunofluorescence analysis of HIF-1α expression in MCF-7 adherent cells and cell clusters (**b**) with mean fluorescence intensity quantification (**c**). *n* = 3. **d**,**e** HIF-1α distribution in lung tissues at 0 h post-injection of 4T1 single cells/clusters (**d**) with positive area quantification (**e**). *n* = 3. **f**,**g** Validation of HIF-1α knockdown efficiency by WB (**f**) with quantification (**g**). *n* = 3. **h** Representative images of CTC clusters formed by MCF-7 cells with or without HIF-1α knockdown. **i** Representative images of cell–cell adhesion within these CTC clusters. **j**,**k** Quantification of cluster area (**j**) and adherent MCF-7-GFP cell number (**k**). *n* = 3. **l** Transmission electron microscopy showing desmosome (yellow arrows) and adherent junctions (AJ) in MCF-7 cell clusters with or without HIF-1α knockdown. *n* = 3. **m** WB analysis of PG, DSG2, E-cadherin and β-catenin expression in MCF-7 cell clusters with or without HIF-1α knockdown. *n* = 3. **n** CTC cluster formation across experimental groups: wild type (Si-Cont), HIF-1α-knockdown (Si-HIF-1α) and rescue (Si-HIF-1α + recombinant DSG2) using MCF-7 cells. *n* = 3. **o** Corresponding intercellular adhesion within CTC clusters. *n* = 3. **p** WB analysis of PG, DSG2, E-cadherin and β-catenin expression in CTC clusters across experimental groups. *n* = 3. Data are mean ± s.d., **P < 0.01, ***P < 0.001.

### Result 5. HIF-1α drives the transition from adhesion junction to desmosome junction through DSG2 to aggravate CTC Clustering

Recognizing that CTC clusters and hypoxic environments are mutually supportive and lead to increased HIF-1α expression^12,15^, we sought to investigate the role of HIF-1α in modulating junctional dynamics during CTC cluster formation. We observed that the expression of HIF-1α and its transcriptional activator P300 in CTC clusters were significantly higher than those in adherent cells (Fig. 5a–c). In a breast cancer lung metastasis model, mice injected with CTC clusters exhibited significantly larger HIF-1α-positive areas in lung tissues compared to those receiving single CTCs (Fig. 5d,e and Supplementary Fig. 5a). Given the known effects of HIF-1α on cell adhesion and DSG2 (ref. ^16^), we hypothesized that HIF-1α might play a major role in regulating cellular junctions in CTC clusters. To test this hypothesis, we successfully knocked down the expression of HIF-1α in MCF-7 cells using siRNA (Fig. 5f,g). The ability of MCF-7 cells to form clusters was significantly weakened after knocking down HIF-1α, along with a significant reduction in cell adhesion (Fig. 5h–k). Consistent with these findings, the HIF-1α knockdown group showed a decrease in cell junctions, especially desmosome junctions (Fig. 5l). The remaining cellular connections were predominantly adhesion junctions (Fig. 5l). Likewise, the knockdown of HIF-1α led to decreased expression of DSG2 and PG, while increasing the expression of E-cadherin and β-catenin compared to the control group (Fig. 5m and Supplementary Fig. 5b). Furthermore, the changes in tumor cell clustering ability, adhesion strength and cell junction protein expression induced by HIF-1α knockdown could be significantly reversed by the addition of recombinant DSG2 (Fig. 5n–p and Supplementary Fig. 5c–e). Taken together, these data highlighted that HIF-1α exacerbates the clustering effect by promoting the transition from adhesion junctions to desmosome junctions via DSG2.

### Result 6. Hirudin inhibits the DSG2-mediated enhancement of desmosome junctions within CTC clusters

**Fig. 6:**
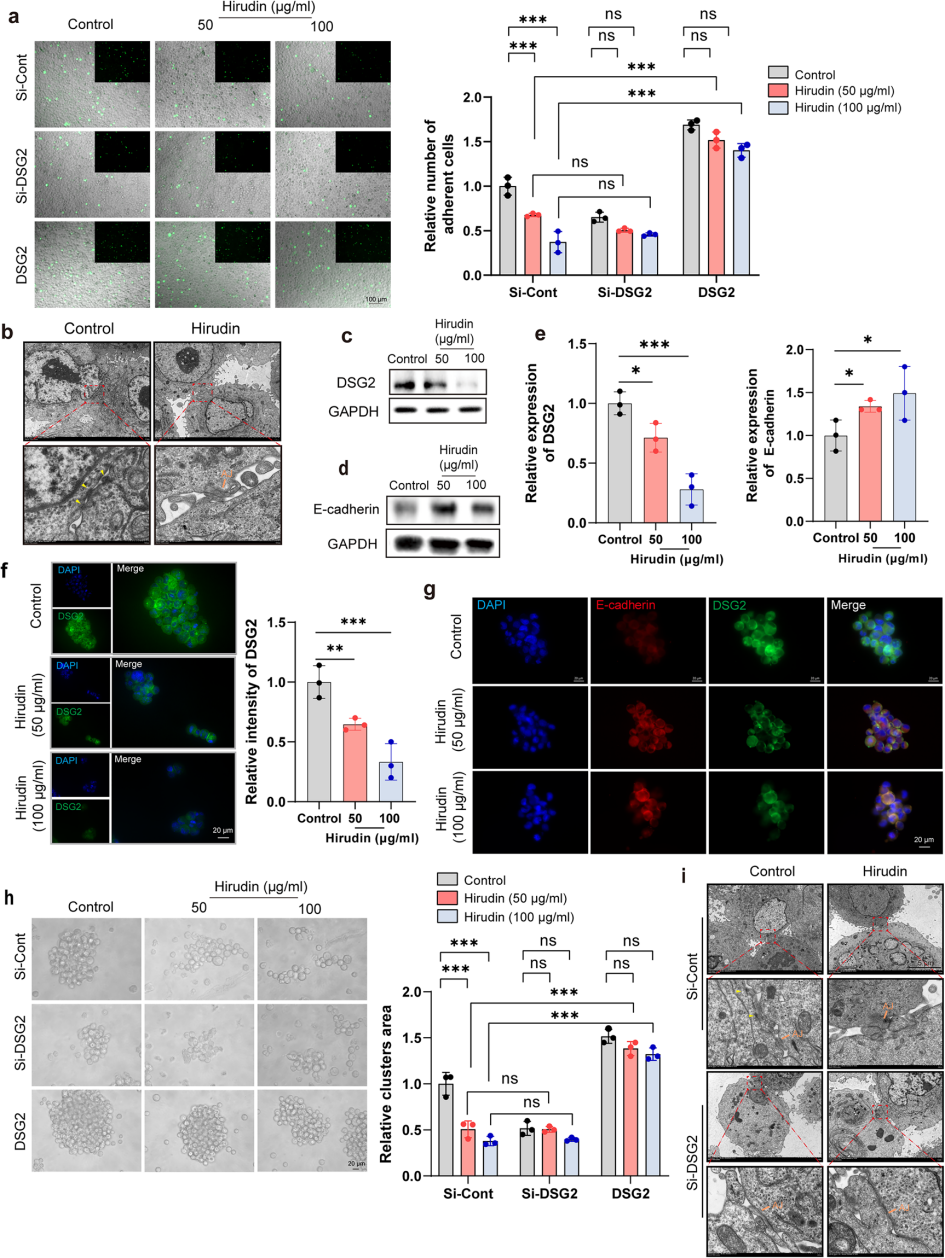
Hirudin promotes the conversion of cellular junctions between CTC clusters from desmosomal junctions to adhesion junctions by inhibiting DSG2. **a** Representative images and quantitative analysis of intercellular adhesion in MCF-7 cell clusters following 24-h hirudin treatment under three conditions: wild type (Si-Cont), DSG2-knockdown (Si-DSG2) and DSG2-reconstituted (+ recombinant DSG2) groups. Upper layer: MCF-7-GFP; lower layer: MCF-7. *n* = 3. Data are mean ± s.d., **P < 0.01. **b** Transmission electron microscopy showing desmosomes (yellow arrows) and adherent junctions (AJ) in MCF-7 cell clusters after 24-h hirudin treatment. *n* = 3. **c**–**e** WB analysis and grayscale quantification of DSG2 and E-cadherin expression in MCF-7 cell clusters following 24-h hirudin treatment. *n* = 3. **f** Immunofluorescence analysis of DSG2 expression in MCF-7 cell clusters after 24-h hirudin treatment, with mean fluorescence intensity quantification. *n* = 3. **g** Immunofluorescence costaining of E-cadherin and DSG2 in MCF-7 cell clusters treated with hirudin for 24 h. *n* = 3. **h** Morphology of CTC clusters formed by DSG2 knockdown or recombinant DSG2-treated MCF-7 cells after 24-h hirudin treatment, with quantification of clusters area percentage. *n* = 3. **i** Transmission electron microscopy images of desmosomes (yellow arrows) and AJ in MCF-7 cell clusters with or without DSG2 knockdown following 24 h of hirudin treatment. *n* = 3. Data are mean ± s.d., *P < 0.05, **P < 0.01, ***P < 0.001.

To elucidate how hirudin inhibits the formation of CTC clusters, we examined its effects on cell adhesion and junctions within these clusters. Hirudin remarkably suppressed the cell adhesion within CTC clusters (Fig. 6a). Electron microscopy showed that hirudin strongly inhibited the formation of desmosome junctions between tumor cells, resulting in looser cell connections compared to untreated clusters (Fig. 6b). As expected, hirudin decreased DSG2 expression while increasing E-cadherin expression, particularly at cell contact sites (Fig. 6c–g). Hirudin also mimicked the effects of DSG2 knockdown (Fig. 6a,h). When DSG2 was knocked down, the inhibitory effect of hirudin on desmosome connection, adhesion and aggregation of breast tumor cells was abolished (Fig. 6a,h,i). Conversely, the addition of recombinant DSG2 protein restored the clustering and adhesion abilities lost due to hirudin treatment (Fig. 6a,h,i). Thus, we suggested that hirudin inhibits CTC clustering is not merely through suppressing cell adhesion, but more critically by blocking the DSG2-mediated strengthening of desmosome junctions within tumor cells.

### Result 7. Hirudin targets HIF-1α to disrupt CTC cluster formation

**Fig. 7:**
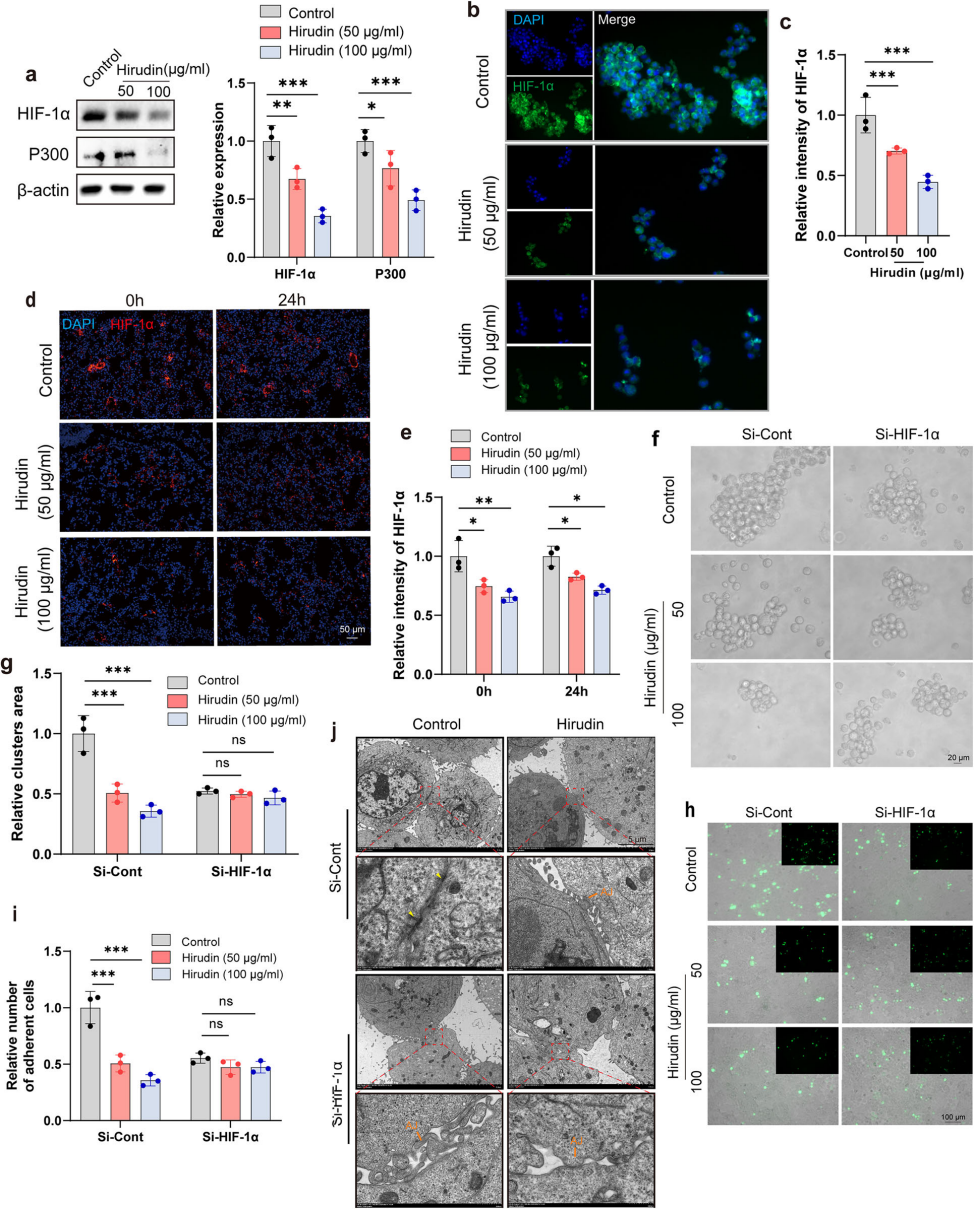
Hirudin promotes the conversion of intercellular junctions in CTC clusters via HIF-1α. **a** WB analysis of HIF-1α and P300 expression in MCF-7 cell clusters after 24-h hirudin treatment, with grayscale quantification. *n* = 3. **b**,**c** Immunofluorescence staining of HIF-1α in MCF-7 cell clusters following 24-h hirudin treatment (**b**) with mean fluorescence intensity quantification (**c**). *n* = 3. **d**,**e** Immunofluorescence images of lung tissue at 0 h and 24 h after tail vein injection of 4T1 cell clusters treated with hirudin or PBS, showing HIF-1α expression (**d**) and quantification of HIF-1α-positive areas (**e**). *n* = 3. **f**,**g** Morphological analysis of CTC clusters formed by control (Si-Cont) and HIF-1α-knockdown MCF-7 cells with or without 24-h hirudin treatment (**f**) with quantification of area percentage (**g**). *n* = 3. **h**,**i** Representative images of intercellular adhesion within CTC clusters (**h**) with MCF-7-GFP cell counts (**i**). Upper layer: MCF-7-GFP; lower layer: MCF-7. *n* = 3. **j** Transmission electron microscopy images of desmosomes (yellow arrows) and adherent junctions (AJ) in MCF-7 cell clusters with or without HIF-1α knockdown following 24 h of hirudin treatment. *n* = 3. Data are mean ± s.d., *P < 0.05, **P < 0.01, ***P < 0.001.

**Fig. 8:**
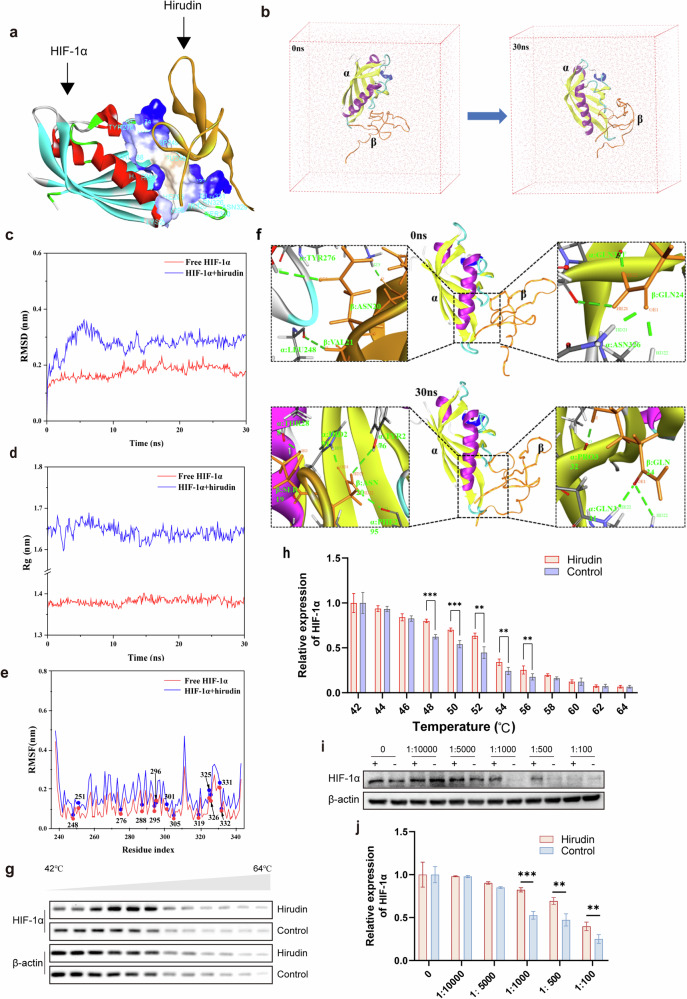
Hirudin binds to HIF-1α. **a** MD analysis of the HIF-1α and hirudin interaction. **b** MD simulation images. Left: 0 ns; right: 30 ns (α: HIF-1α; β: hirudin). **c** RMSD plot. **d** Radius of gyration (*R*_g_) plot. **e** RMSF plot of HIF-1α amino acid residues. **f** Hydrogen bond interaction analysis between HIF-1α and hirudin at 0 ns and 30 ns. **g**,**h** CETSA assay showing the binding of hirudin and HIF-1α (**g**) with grayscale quantification (**h**). *n* = 3. **i**,**j** DARTS assay confirming the hirudin–HIF-1α interaction (**i**) with grayscale quantification (**j**). *n* = 3. Data are mean ± s.d., **P < 0.01, ***P < 0.001 versus control.

Following our identification that HIF-1α promotes desmosome junctions through DSG2 in CTC clusters and that hirudin disrupts these junctions, we next investigated whether hirudin directly modulates HIF-1α. WB and immunofluorescence analyses showed that hirudin markedly suppressed the expression levels of HIF-1α and P300 in in vitro CTC clusters (Fig. 7a–c). Moreover, the expression of HIF-1α was significantly reduced in the hirudin-treated cell clusters group during both lung retention (Fig. 7d,e) and colonization (Supplementary Fig. 5a). However, when HIF-1α was knocked down, hirudin lost its inhibitory effects on breast tumor cell aggregation, adhesion and desmosome junction formation (Fig. 7f–j). Therefore, HIF-1α, which is upstream of DSG2, may be the target of hirudin in CTC clusters disruption. To prove this conjecture, we first utilized molecular docking to examine the possibility of hirudin binding to HIF-1α and found that hirudin could form strong hydrogen bonds and hydrophobic interactions with HIF-1α residues (Fig. 8a). The binding interface contained amino acid residues such as Ser247, Leu248, Asp249 and Met250, which are mainly hydrophilic (Fig. 8a). To further illustrate the structural stability of the HIF-1α–hirudin complex, we performed 30 ns MD simulations. The data showed that the active binding pocket and binding region of the complex remained unchanged throughout the simulation (Fig. 8b). Although the root mean square deviation (RMSD) and radius of gyration (*R*_g_) of the HIF-1α–hirudin complex were higher than those of HIF-1α alone, these values stabilized after 10 ns, suggesting that the complex maintained a relatively stable structure (Fig. 8c,d). The root mean square fluctuations (RMSF) of HIF-1α exhibited increased variability when complexed with hirudin, highlighting differences in the flexibility of HIF-1α residues (Fig. 8e). Key residues for the stable interaction between hirudin and HIF-1α included Leu248, Tyr276, Asn326, Gln331 and Pro332 (Fig. 8e,f and Supplementary Table 2). To verify the direct interaction between hirudin and HIF-1α, we initially confirmed the presence of hirudin in the cytoplasm (Supplementary Fig. 6aand Supplementary Table 3). Subsequently, we detected a hirudin band in HIF-1α immunoprecipitates, with HPLC–MS/MS showing 100% coverage of three unique hirudin peptides, providing strong evidence for the interaction (Supplementary Fig. 6b,c and Supplementary Table 4). Furthermore, CETSA and DARTS assays also demonstrated the direct binding of hirudin to HIF-1α (Fig. 8g–j).

**Fig. 9:**
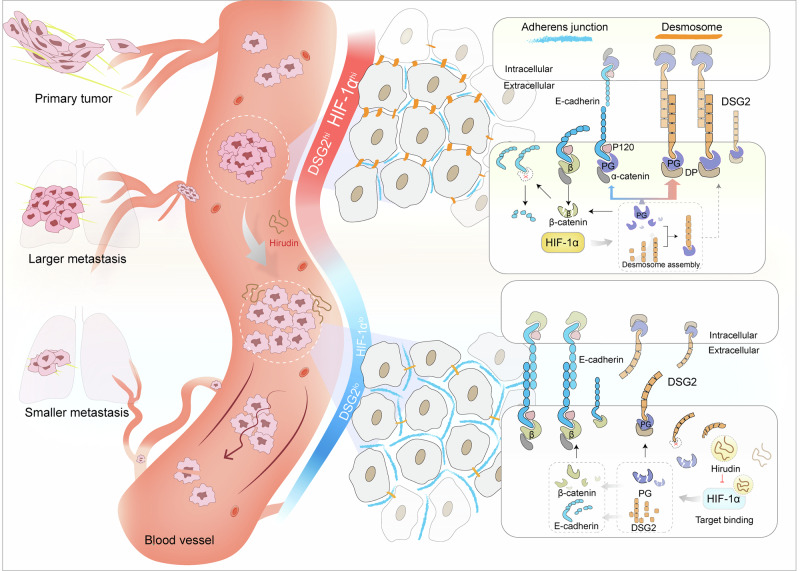
Mechanistic diagram of hirudin inhibiting lung metastasis of breast tumor clusters.

In summary, the anticoagulant hirudin binds to HIF-1α and downregulates its expression. This action disrupts DSG2-mediated desmosome junctions and weakens cell adhesion, thereby inhibiting the formation of CTC clusters in breast tumor cells and reducing lung metastasis (Fig. 9).

## Discussion

Tumor metastasis involves multiple cascading processes, with CTCs regarding as the basic unit of tumor dissemination. However, CTCs are not only present in the peripheral blood as single cells but also aggregate into multicellular populations. The metastasis of clustered CTCs is more efficient than single cells and is associated with lower overall survival in patients with breast tumors^2^. Our study reached a similar conclusion, observing more significant lung metastasis in the CTC cluster group compared to the single-cell group. Although the exact site of CTC cluster formation remains controversial, adhesion is considered an important factor in their formation and transfer^4,33,34^. The maintenance of structural integrity and survival of CTC clusters in circulation fundamentally relies on the high expression of intercellular adhesion proteins^29^. Traditionally, anticoagulant drugs are used to prevent and treat thrombotic diseases by inhibiting key factors in the blood coagulation system. However, recent evidence suggests anticoagulants may also reduce the formation and retention of tumor emboli in the microvasculature to lower tumor metastatic potential^18^. Several drugs with anticoagulant properties, such as aspirin, have been revealed to be useful for tumor prevention^19,20^. Thrombolytic agents such as urokinase can disrupt cell aggregates and decrease the incidence of CTC clusters^22^. In our study, we found that both heparin and hirudin inhibit lung metastasis of breast tumor cells. Moreover, hirudin specifically inhibits the formation of CTC clusters and reduces their metastatic potential by decreasing their adhesiveness. Nevertheless, anticoagulants are prone to triggering bleeding, so it is important to confirm the safe dosage of such drugs before using them in tumor therapy.

The expression of adhesion proteins and the formation of intercellular connections are two key processes of cell aggregation. Intercellular connections maintain the morphology of CTC clusters in blood vessels^16^. Therefore, cell aggregation, especially of CTC clusters, cannot be limited to tumor cell adhesion, but also needs to focus on a more subtle level of how tumor cells are connected. In addition to the adhesion junction, desmosomes are another type of intercellular junction with high adhesion between epithelial cells. Desmosomes include two calmodulins, desmosome granule adhesions (DSGs) and desmosome granule adhesion protein (DSCs)^35,36^. Among them, DSG2 is a key factor in breast cell aggregation and carcinogenesis. In our study, DSG2 expression was markedly elevated in breast cancer tumor tissues relative to adjacent nontumor tissues. Moreover, higher levels of DSG2 were detected in cell clusters than in individual cells from patient blood samples. It has been proposed that subpopulations of cells with greater metastatic capacity have higher expression of adhesion proteins^37^. Consistent with this, CTC clusters captured from tumor patients and tumor model mice exhibited high adhesion protein expression^2^. We speculated that this may be related to the need for higher adhesion to maintain their cluster morphology during hematogenous metastasis. However, other studies have shown that the downregulation of the adhesion junction component E-cadherin and the desmosome components DSG2 and DSC2 is associated with increased tumor metastasis and poor prognosis^38^. Although various models have been tested to explain why the downregulation of adhesion proteins promotes tumor metastasis, these models primarily apply to cell invasion processes. Thus, the relationship between cell adhesion and metastasis deserves to be revisited in the context of cell clusters, which serve as precursors to metastasis.

Cells construct stronger cellular junctions by assembling a large number of desmosomes after forming weak adhesion junctions^39^. PG is the only protein shared between these two types of cell junctions^40^. Furthermore, PG is highly expressed in CTC clusters compared to single CTCs^2^. Downregulation of DSC2 will lead to the redistribution of adherens junction molecules^41^. Thus, there may be crosstalk between these two types of connections in CTC clusters. Downregulation of E-cadherin in situ tumor cells promotes cell detachment and entry into the blood circulation. However, the increased expression level of E-cadherin in CTC clusters compared to single CTCs suggests a different role for E-cadherin in tumor metastasis^42,43^. Interestingly, unlike these studies, our observations showed that CTC cluster DSG2 expression was significantly elevated, and E-cadherin was significantly decreased compared to adherent cells representing primary tumor tissues. As CTC clusters dissociated, stronger desmosome connections weakened, and weaker adherent connections became more prominent. Although our control cells were adherent and not individual CTCs, our findings aligned with previous studies showing that CTCs connect and adhere better when clustered. More importantly, we revealed that the maintenance of CTC clusters was more dependent on DSG2-mediated desmosome junctions than E-cadherin-mediated adhesion junctions. However, whether there are differences in the expression of E-cadherin in different CTC subpopulations and different metastatic segments still needs to be further investigated. The regulatory role of E-cadherin in signaling pathways like Wnt/β-catenin, PI3K/Akt and NF-κB—which are implicated in cell proliferation, differentiation and immune response—should not be overlooked^44^. As part of the adhesion junction complex, the stability and localization of β-catenin are regulated by GSK-3β. Undegraded β-catenin accumulates and translocates to the nucleus, activating LEF/TCF transcription factors and modulating the expression of cell cycle-related genes such as cyclin D1 and c-myc^45^. In our study, E-cadherin expression was increased after hirudin administration. To better explain the antimetastatic mechanism of hirudin, the interference of signal transduction needs to be excluded.

Hypoxia is a characteristic of CTC clusters^15^. Our study confirmed that HIF-1α promoted DSG2 expression in cell clusters and was a regulatory protein for DSG2-mediated cell junctional transitions. However, Chang et al.^16^ found that increased HIF-1α in hypoxic regions of human breast tumors inhibits DSG2 expression, promoting the formation of CTCs. Once in the bloodstream, cells release from hypoxic stress, leading to a reduction of HIF-1α levels and restoration of DSG2 expression, which promotes tumor cell clustering^16^. Spatial differences in oxygen levels within primary tumors and tumor heterogeneity result in some in situ tumor samples showing concurrent low expression of HIF-1α and DSG2^16^. Unfortunately, their findings do not explain how CTC clusters maintain DSG2 expression upon activation of HIF-1α. It is possible that HIF-1α flexibly regulates adhesion proteins such as DSG2 at different stages of tumor metastasis. However, how the oxygen level changes in different states of tumor cells in the bloodstream metastasis needs further study.

In our study, we revealed the cellular connectivity mode of CTC clusters and their crosstalk mechanism, while confirming the antimetastatic effect of hirudin in breast cancer. We discovered that hirudin inhibits tumor cell clustering by impairing DSG2-mediated desmosome through binding to HIF-1α, although the specific binding site remains unvalidated. MD simulation suggests residues such as Tyr276 were involved in complex binding, which requires further validation through techniques such as point mutation. Beyond homotypic clusters, CTCs also form heterotypic aggregates with immune cells (for example, neutrophils) and platelets, which cooperatively enhance metastatic potential through distinct mechanisms^46,47^. Neutrophils release neutrophil extracellular traps (NETs) that facilitate CTC extravasation, while platelets provide physical protection and biochemical activation of EMT via TGFβ1 (refs. ^48,49^). Although isolating heterotypic clusters is more challenging, their investigation is essential for comprehensive evaluation of the antimetastatic effect of hirudin. Future studies should focus on developing methods to capture and analyze heterotypic CTC clusters to better understand their role in metastasis and the potential impact of hirudin.

During metastasis, tumor cells dynamically adapt their morphology in response to microenvironmental cues. CTC clusters can rapidly and reversibly reorganize into single-stranded cells to traverse microvessels, reducing hemodynamic resistance^50^. Platelet-derived TGFβ1 also promotes cluster dissociation into individual cells for vascular wall adhesion^51^. Single CTCs dissociated from clusters may retain cluster-acquired properties, such as enhanced stemness and proliferative capacity, which could potentiate metastatic outgrowth. While our study elucidated the disruptive effects of hirudin on CTC clusters, its impact on single CTCs, particularly those carrying residual cluster-derived traits, remains unexplored. Experimental strategies including single-cell RNA-seq and microfluidic isolation should be employed in the future to systematically compare the molecular profiles and metastatic competence of cluster-dissociated versus spontaneous single CTCs, which further refines the antimetastasis profile of hirudin.

## Supplementary information

Supplementary Information

## Data Availability

The datasets used during the current study are available from the corresponding author upon reasonable request.
