# Supplementary material for: Hirudin suppresses hematogenous metastasis by targeting desmosome junction transition in circulating tumor cell clusters via HIF-1α–DSG2 signaling
Source: Exp Mol Med. 2025 Dec 12;57(12):2798–815. doi: 10.1038/s12276-025-01598-8 (PMC12800233; doi:10.1038/s12276-025-01598-8)
Supplement: Supplementary file 1 — Supplementary Information [file 12276_2025_1598_MOESM1_ESM.pdf]

Supplementary materials

Cell Apoptosis Analysis

Apoptosis was assessed using an Annexin V-FITC/PI Apoptosis Detection Kit (40302ES60, Yeasen, China). Cells were stained with Annexin V-FITC and propidium iodide (PI) for 15 minutes, followed by fluorescence microscopy analysis (Zeiss Axio Vert A1 microscope). The apoptosis rate is calculated as the proportion of apoptotic cells within the total cell population.

3D Invasion Assay of Cell Clusters

After 24-hour hirudin treatment (0, 50, or 100 μg/mL), MCF-7 cell clusters were cultured in serum-free DMEM for an additional 24 hours The cell clusters were then embedded in Corning Matrigel Basement Membrane Matrix (354234, Corning, USA) diluted at a 1:2 ratio with serum-free DMEM medium. After the matrix solidified, differential interference contrast (DIC) images were captured every 30 minutes at 40× magnification using an IncuCyte Zoom (Essen Bioscience, USA).

Immunoprecipitation

MCF-7 cell clusters were treated with 100 μg/mL hirudin for 24 hours. Immunoprecipitation was performed using an Immunoprecipitation Kit with Protein A+G Agarose Gel (P2179S, Beyotime, China), HIF-1α Rabbit mAb (36169T, Cell Signaling Technology, USA), and BeyoMag™ Magnetic Separation Rack (FMS008, Beyotime, China). Briefly, cells were lysed and centrifuged at 10,000g for 5 min at 4°C to collect the supernatant. The supernatant was incubated with either HIF-1α antibody or IgG control antibody overnight at 4°C, followed by the addition of magnetic beads and incubation at room temperature for 1 h. After magnetic separation and washing,

bound proteins were eluted with a loading buffer at 95°C for 5 min. The immunoprecipitated samples were analyzed by Western blotting using a Hirudin Rabbit pAb (bs-9508R, Bioss, China).

HPLC-MS/MS Analysis

After treating MCF-7 cell clusters with 100 μg/mL hirudin for 24 hours, IgG and HIF-1α immunoprecipitates were isolated, separated by SDS-PAGE, and bands around 7 kDa were excised for hirudin detection via HPLC-MS/MS. The gel pieces were destained, dehydrated, and dried under vacuum, then reduced with DTT, alkylated with IAM, and digested with trypsin overnight. The supernatant was collected by centrifugation, transferred to a new tube, dried under vacuum, desalted, and reconstituted in 0.1% formic acid. The peptides were analyzed using an EASY-nLC 1200 UPLC system coupled to a Thermo Q Exactive HF-X mass spectrometer. Mobile phase A was 0.1% formic acid in water, while mobile phase B was 0.1% formic acid in acetonitrile. The elution gradient was as follows: 3%–5% B (5 s), 5%–15% B (23 min 55 s), 15%–28% B (21 min), 28%–38% B (7 min 30 s), 38%–100% B (5 s), and 100% B (12 min 25 s). The flow rate was maintained at 300 nL/min. The ion source voltage was set at 2 kV. Both precursor ions and their secondary fragments were detected using the high-resolution Orbitrap. Scan range: 350-1500 m/z. MS1 resolution: 60,000. MS2 resolution: 15,000. Data acquisition was in DDA mode with AGC targets of 3e6 for MS1 and 1e5 for MS2, and dynamic exclusion of 30 s. Raw data were processed using Proteome Discoverer (v2.4) with the following parameters: the database used was a combination of uniprot_human and the hirudin amino acid sequence (named: Hirudinsequence); the digestion enzyme was Trypsin/P; the number of missed cleavage

sites was 2; the minimum peptide length was 6 amino acid residues; cysteine alkylation was a fixed modification; methionine oxidation and protein N-terminal acetylation were variable modifications.

For the detection of hirudin in cytoplasmic fractions of MCF-7 cell clusters, DIA quantitative proteomics was employed. Samples were lysed in 8 M urea buffer, mixed thoroughly, and sonicated. Lysates were incubated on ice for 30 min with intermittent vortexing and centrifugated at 14,000g for 30 min at 4 °C. The supernatant was subjected to reduction with DTT, alkylation with IAM, and digestion with trypsin (1:100 enzyme: protein ratio) 16h at 37 °C. The resulting peptides were desalted, lyophilized, and reconstituted in 0.1% formic acid for analysis by the EASY-nLC 1200 UPLC system. Mobile phases were 0.1% formic acid in water (A) and acetonitrile (B). Peptides were eluted with a gradient of 3%–5% B (5 s), 5%–15% B (40 min), 15%–28% B (34 min 5 s), 28%–38% B (12 min), 38%–100% B (5 s), and 100% B (8 min) at 300 nL/min. Mass spectrometry analysis was performed on a Thermo Scientific Q Exactive™ HF-X instrument with an ion source voltage of 2.2 kV. DIA was used with full MS scans at 400−1250 m/z (120,000 resolution) and MS/MS scans at 30,000 resolution. An isolation window of 18 m/z was used, with AGC set to 1 1e6 and maximum injection time in auto mode. Data were processed using DIA-NN (v1.9.1) against a combined database of uniprot_human and the hirudin sequence. Search parameters included Trypsin/P digestion with up to two missed cleavages, peptide length of 7-30 amino acids, and fixed carbamidomethylation of cysteine residues.

Supplementary figures and legends

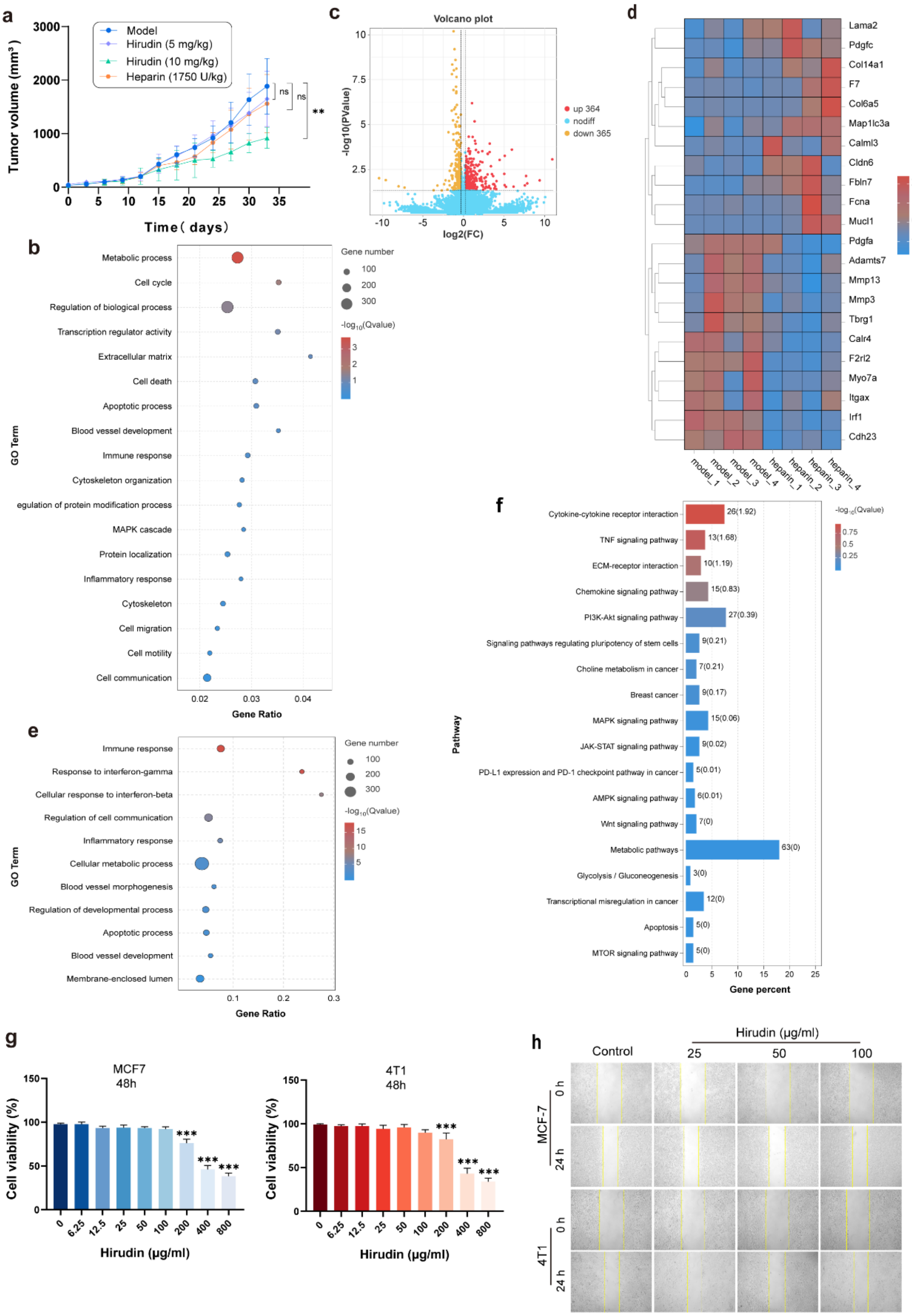
a
Model
Hirudin (5 mg/kg)
Hirudin (10 mg/kg)
Heparin (1750 U/kg)
Tumor volume (mm³)
Time (days)
ns
ns
**
b
Gene number
-log10(Qvalue)
GO Term
Gene Ratio
Metabolic process
Cell cycle
Regulation of biological process
Transcription regulator activity
Extracellular matrix
Cell death
Apoptotic process
Blood vessel development
Immune response
Cytoskeleton organization
egulation of protein modification process
MAPK cascade
Protein localization
Inflammatory response
Cytoskeleton
Cell migration
Cell motility
Cell communication
c
Volcano plot
-log10(PValue)
log2(FC)
up 364
nodiff
down 365
d
Lama2
Pdgfc
Col14a1
F7
Col6a5
Map1lc3a
Calml3
Cldn6
Fbln7
Fcna
Mucl1
Pdgfa
Adamts7
Mmp13
Mmp3
Tbrg1
Calr4
F2rl2
Myo7a
Itgax
Irf1
Cdh23
model_1
model_2
model_3
model_4
heparin_1
heparin_2
heparin_3
heparin_4
e
Gene number
-log10(Qvalue)
GO Term
Gene Ratio
Immune response
Response to interferon-gamma
Cellular response to interferon-beta
Regulation of cell communication
Inflammatory response
Cellular metabolic process
Blood vessel morphogenesis
Regulation of developmental process
Apoptotic process
Blood vessel development
Membrane-enclosed lumen
f
Pathway
Gene percent
-log10(Qvalue)
Cytokine-cytokine receptor interaction 26(1.92)
TNF signaling pathway 13(1.68)
ECM-receptor interaction 10(1.19)
Chemokine signaling pathway 15(0.83)
PI3K-Akt signaling pathway 27(0.39)
Signaling pathways regulating pluripotency of stem cells 9(0.21)
Choline metabolism in cancer 7(0.21)
Breast cancer 9(0.17)
MAPK signaling pathway 15(0.06)
JAK-STAT signaling pathway 9(0.02)
PD-L1 expression and PD-1 checkpoint pathway in cancer 5(0.01)
AMPK signaling pathway 6(0.01)
Wnt signaling pathway 7(0)
Metabolic pathways 63(0)
Glycolysis / Gluconeogenesis 3(0)
Transcriptional misregulation in cancer 12(0)
Apoptosis 5(0)
MTOR signaling pathway 5(0)
g
MCF7
48h
4T1
48h
Cell viability (%)
Hirudin (μg/ml)
***
h
Control
Hirudin (μg/ml)
25
50
100
MCF-7
4T1
0 h
24 h

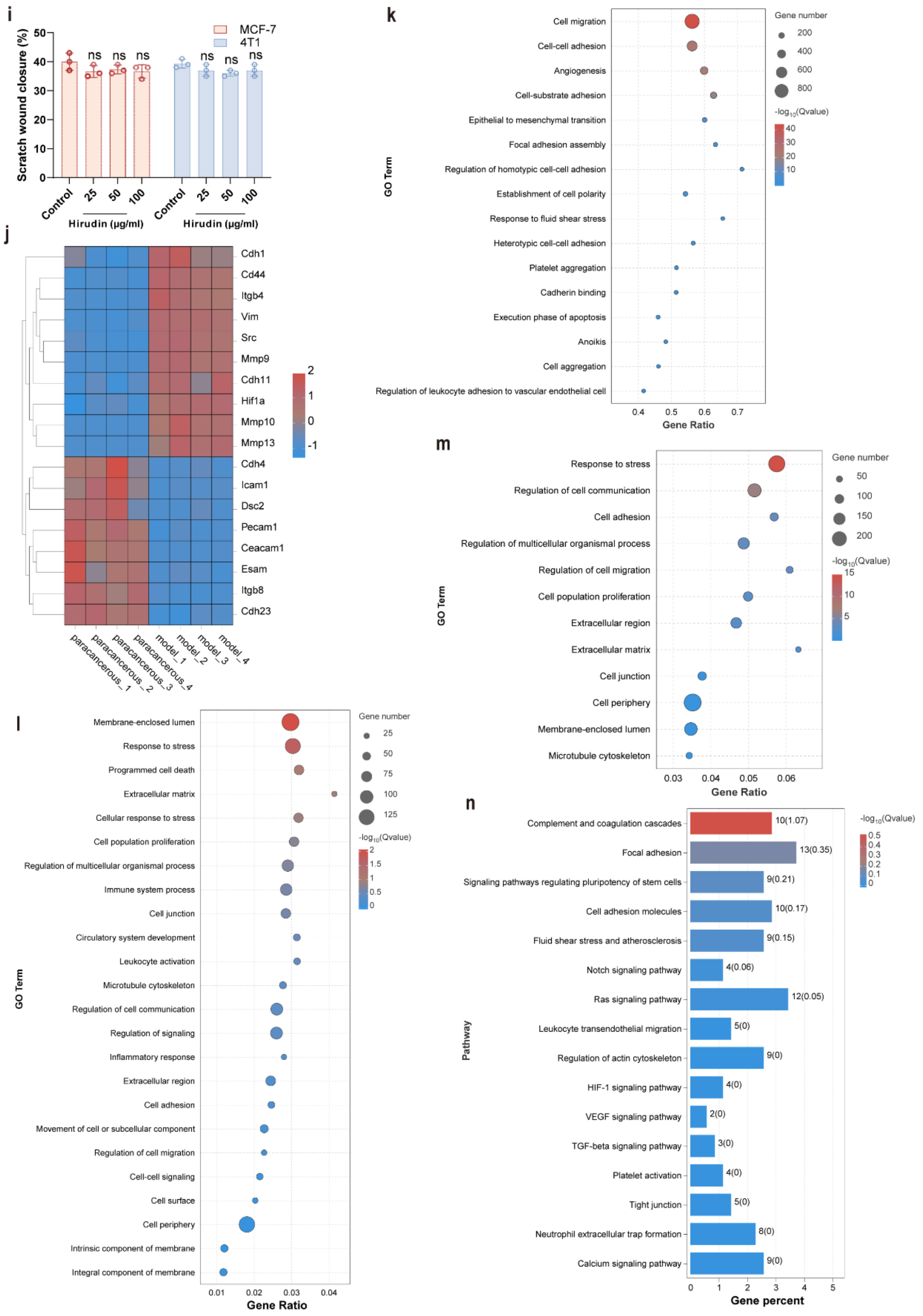

Supplementary Fig. 1. Hirudin suppresses the lung metastasis of breast tumor cells.

(a) The tumor volume was measured every 3 days. n= 7. (b) GO enrichment analysis of differential genes in primary tumor tissues from the model and 5 mg/kg hirudin groups. n= 4. (c) The volcano plot of differential genes from primary tumor tissue of the model

group and heparin group. (d) The heat map displays significantly different metastasis-related genes in primary tumor tissues of the model and heparin groups. (e-f) GO enrichment analysis and KEGG enrichment analysis of differential genes in primary tumor tissues from the model and heparin groups. n= 4. (g) The cell viability of MCF-7 cells and 4T1 cells after 48-hour treatment with hirudin. n= 6. (h-i) The wound healing assay was used to determine the migration of MCF-7 and 4T1 cells after 24-hour treatment with hirudin. n=3. (j-k) The heatmap and GO Term of differential genes associated with the formation of CTCs in the paraneoplastic and tumor tissue of model mice. GO enrichment analysis of differential genes in primary tumor tissues of model and 5 mg/kg hirudin groups, and model and heparin groups, where GO term related to CTC clusters formation were presented in (l) and (m), respectively. (n) Pathways associated with the formation of CTC clusters in KEGG enrichment analysis of differential genes in the model and heparin groups. n=4. Data are mean±SD, $^{**}P<0.01$, $^{***}P<0.001$.

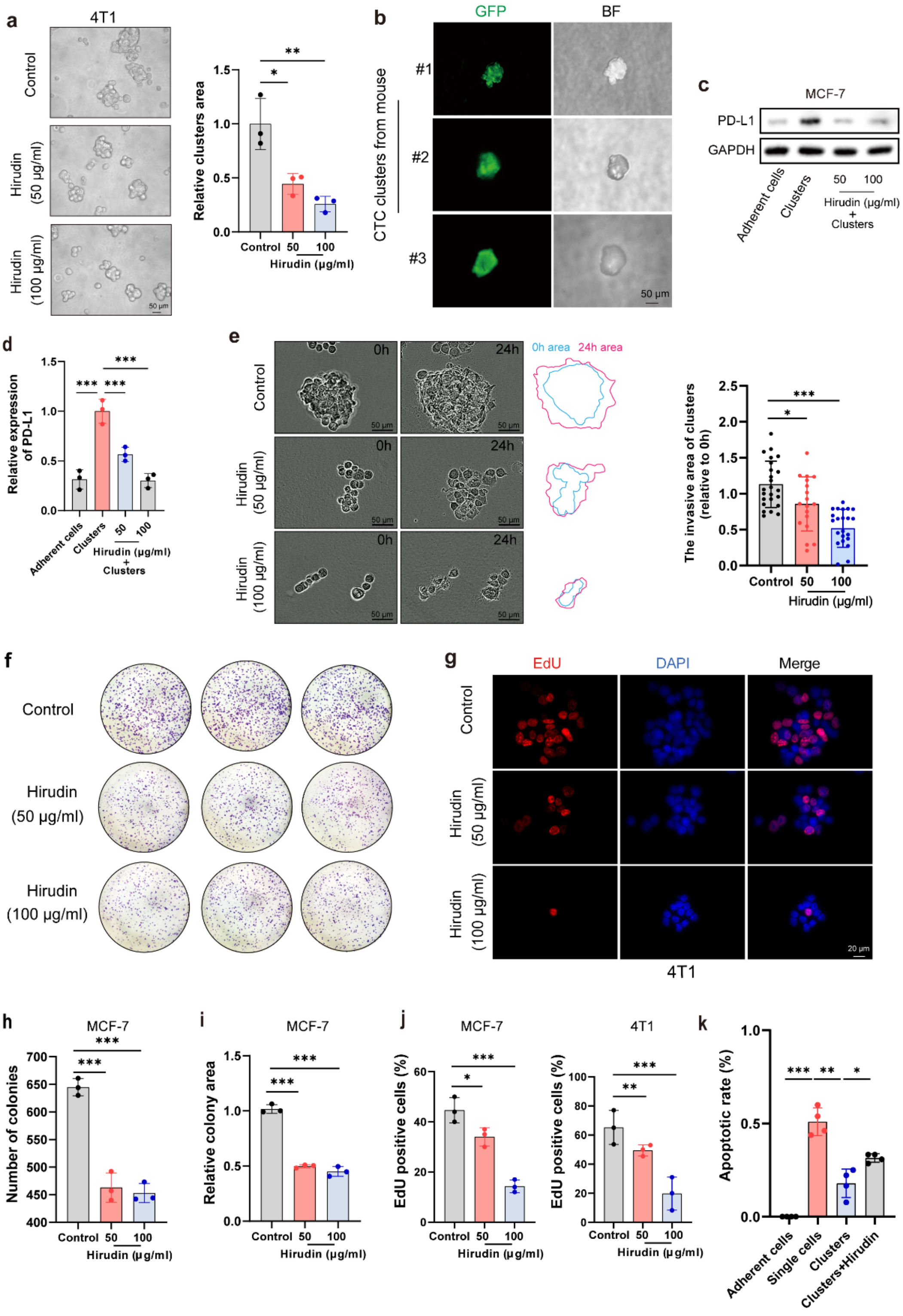

a
4T1
Control
Hirudin (50 μg/ml)
Hirudin (100 μg/ml)
Relative clusters area
Control 50 100
Hirudin (μg/ml)
b
GFP
BF
CTC clusters from mouse
#1
#2
#3
50 μm
c
MCF-7
PD-L1
GAPDH
Adherent cells
Clusters
50 100
Hirudin (μg/ml) + Clusters
d
Relative expression of PD-L1
Adherent cells
Clusters
Hirudin (μg/ml) + Clusters
e
0h
24h
0h area 24h area
The invasive area of clusters (relative to 0h)
Control 50 100
Hirudin (μg/ml)
f
Control
Hirudin (50 μg/ml)
Hirudin (100 μg/ml)
g
EdU
DAPI
Merge
20 μm
4T1
h
MCF-7
Number of colonies
Control 50 100
Hirudin (μg/ml)
i
MCF-7
Relative colony area
j
MCF-7
4T1
EdU positive cells (%)
k
Apoptotic rate (%)
Adherent cells
Single cells
Clusters
Clusters+Hirudin

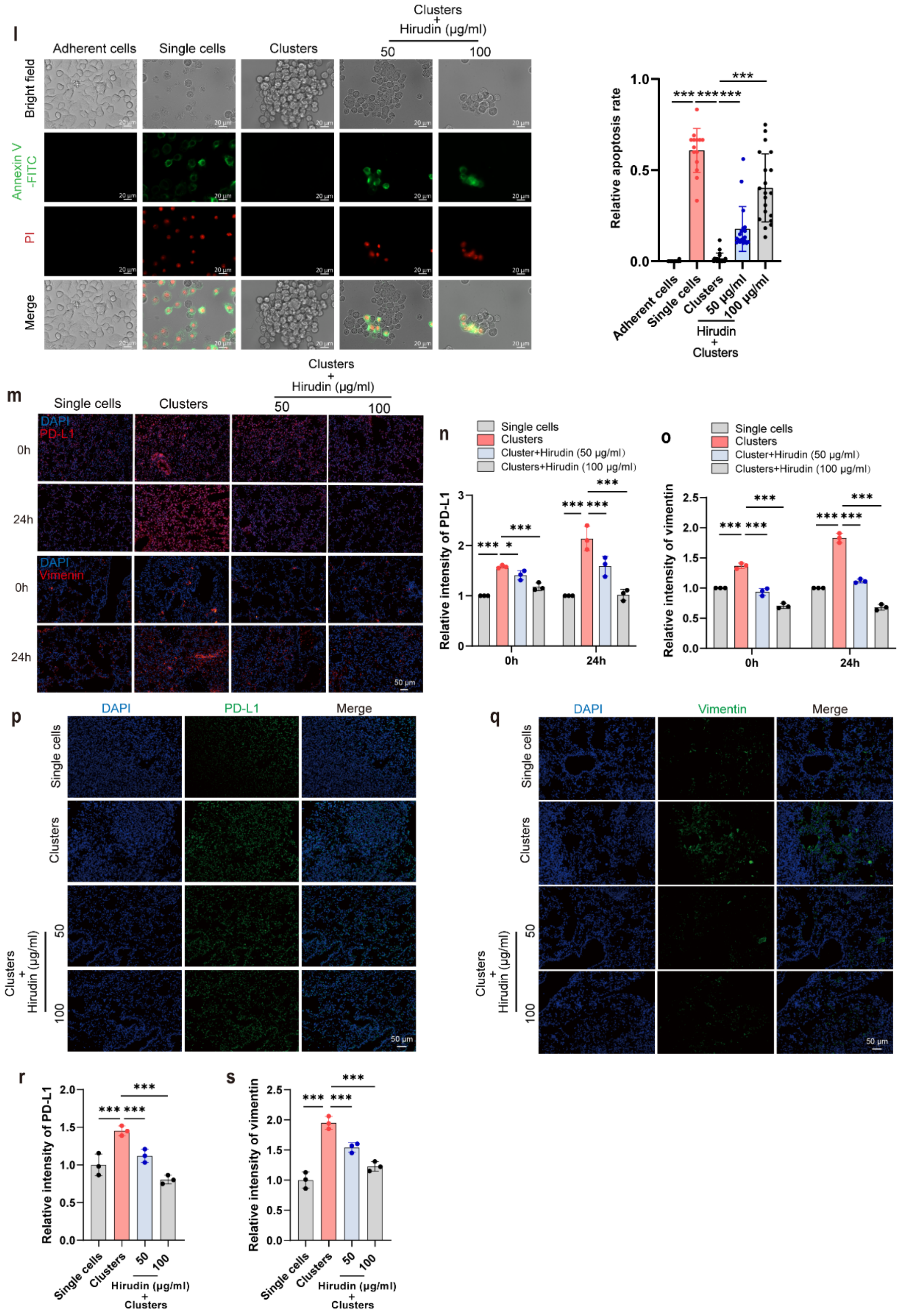

Supplementary Fig. 2. Hirudin weakens the characteristics of CTC clusters.

(a) Morphology of 4T1 clusters constructed in vitro. (b) Morphology of CTC clusters in peripheral blood of 4T1 orthotopic transplanted tumor mice (Diameter > 20μm). n=3. (c-d) The protein expression of PD-L1 after 24 h treatment of hirudin or PBS. n=3. (e)

The invasion ability of MCF-7 cell clusters treated with or without hirudin for 24 hours. The invasion area was quantified. n=3. (f-j) CTC clusters were blown apart into single cells, and the proliferation of tumor cells within the clusters was examined by cloning assay and conformed by Edu staining. (f, h-j) MCF-7 cell clusters. (g) 4T1 cell clusters. Quantitative analysis of the number of colonies, colony area, and Edu-positive cells was shown in (h-j). n=3. (k) Quantitative analysis of the apoptotic rate in transmission electron micrographs. n=4. (l) Analysis of apoptosis of MCF-7 cells in different states. CTC clusters were treated with or without hirudin for 24 hours. n=3. (m-s) Fluorescent staining images of PD-L1 and Vimentin in murine lung tissue at 24 h (m-o) and 14 days (p-s) after 4T1-luc cell modeling. n=3. Data are mean±SD, $*P<0.05$, $**P<0.01$, $***P<0.001$.

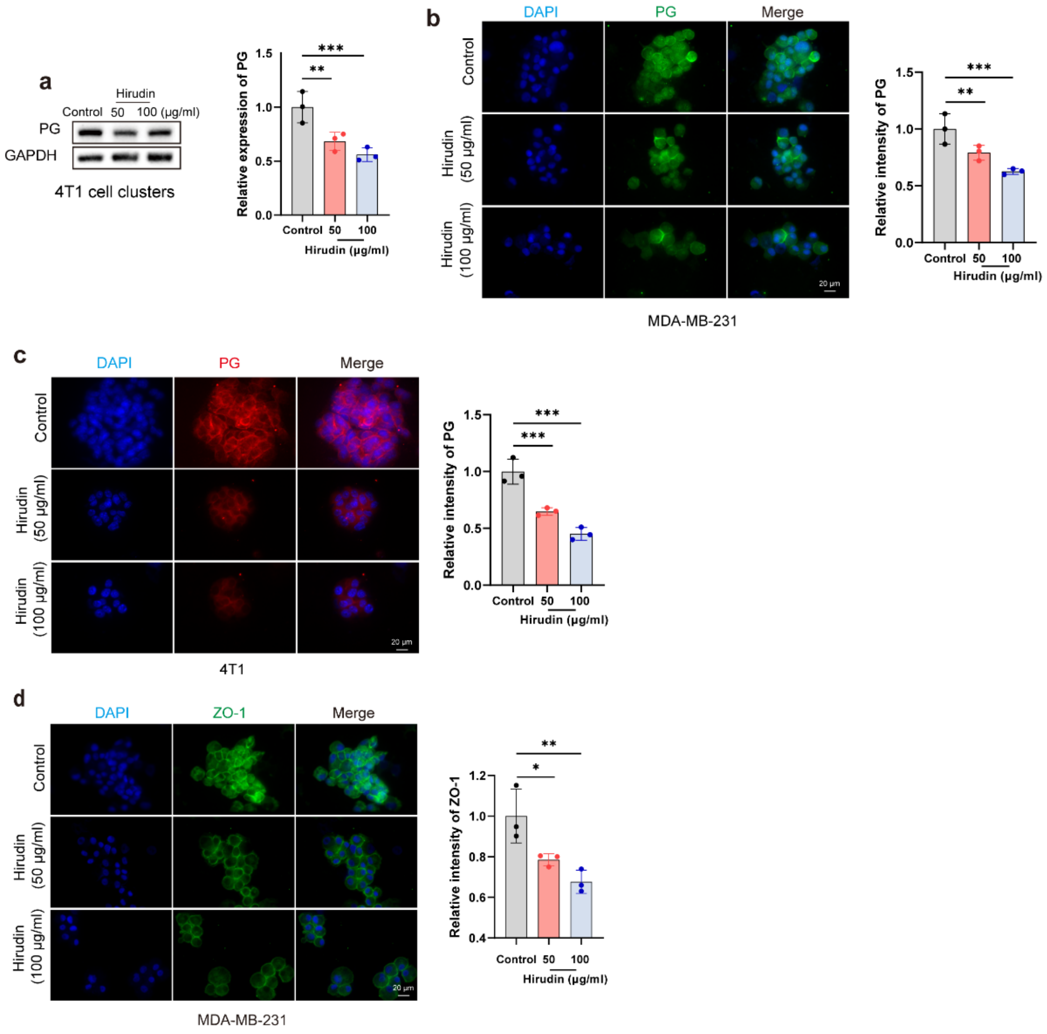

Supplementary Fig. 3. Hirudin decreases the expression of adhesion protein in breast tumor cell clusters.

(a) WB analysis of PG protein expression in 4T1 cell clusters after 24-hour hirudin treatment, with quantification of grayscale values. n = 3. (b-c) Immunofluorescence staining showing PG protein expression and localization in (b) MDA-MB-231 cell clusters and (c) 4T1 cell clusters treated with or without hirudin for 24 hours. Mean fluorescence intensity was quantified. n = 3. (d) Immunofluorescence analysis of ZO-1 protein expression and localization in MDA-MB-231 cell clusters following 24-hour hirudin treatment, with mean fluorescence intensity quantification. n = 3. Data are mean±SD, *P<0.05, **P<0.01, ***P<0.001.

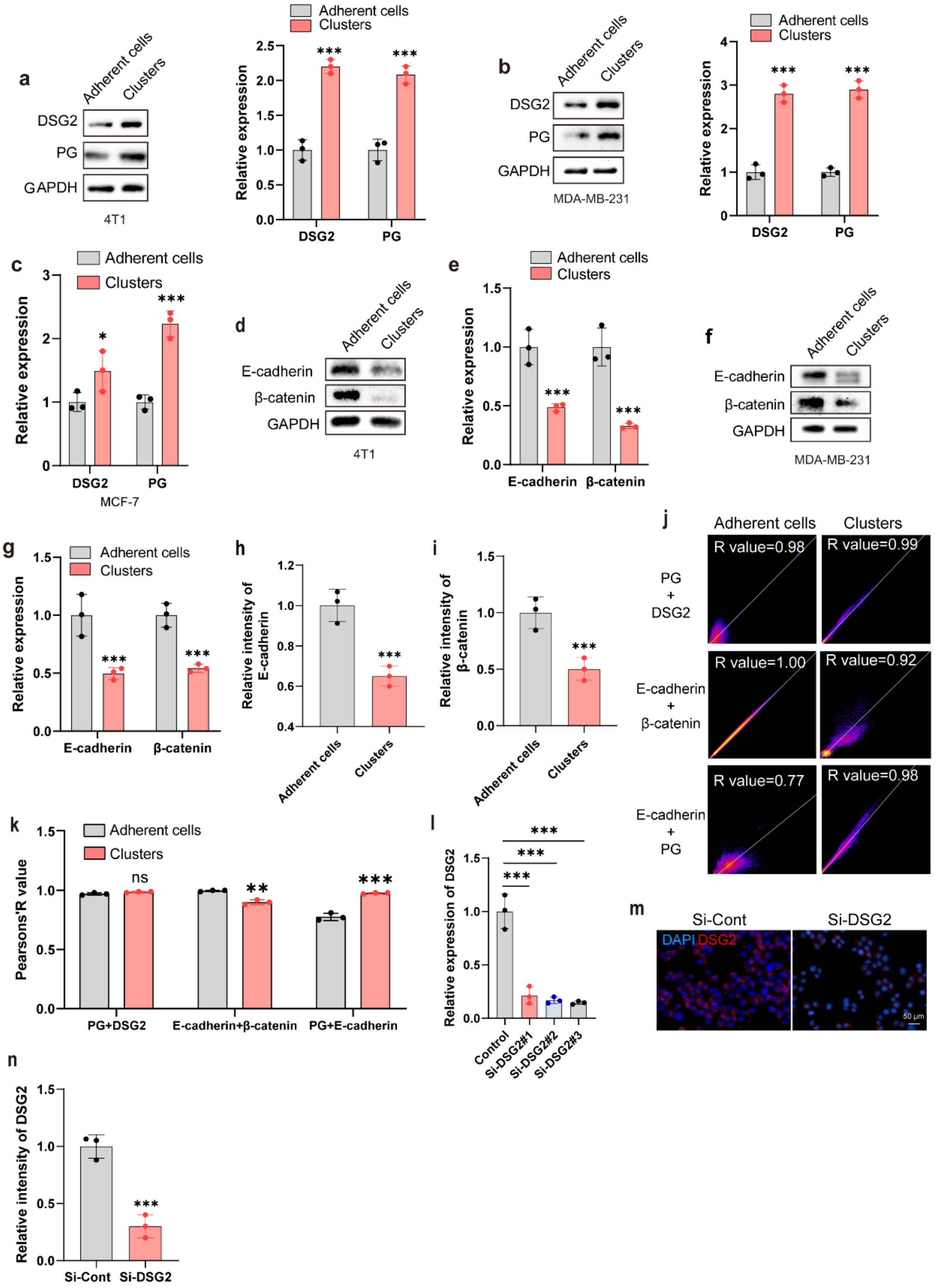

Supplementary Fig. 4. Desmosomes are the main type of junction between cells in 4T1 and MDA-MB-231 cell clusters.

(a-g) Protein expression and quantitative analysis of DSG2, PG, E-cadherin, and β-catenin in adherent cells and cell clusters. (a, d, e) 4T1 cells, (b, f, g) MDA-MB-231

cells. (c) MCF-7 cells. n = 3. (h-i) Quantitative analysis of the relative intensity of (h) E-cadherin and (i) β-catenin in MCF-7 adherent cells and clusters. (j-k) Colocalization analysis of PG and DSG2, E-cadherin and β-catenin, E-cadherin and PG proteins. (l) Quantitative analysis of DSG2 protein expression in cells treated with DSG2 siRNA. (m-n) Immunofluorescence analysis of the knockdown effect of DSG2. The relative intensity is shown in plot (n). n = 3. Data are mean±SD, **P<0.01, ***P<0.001.

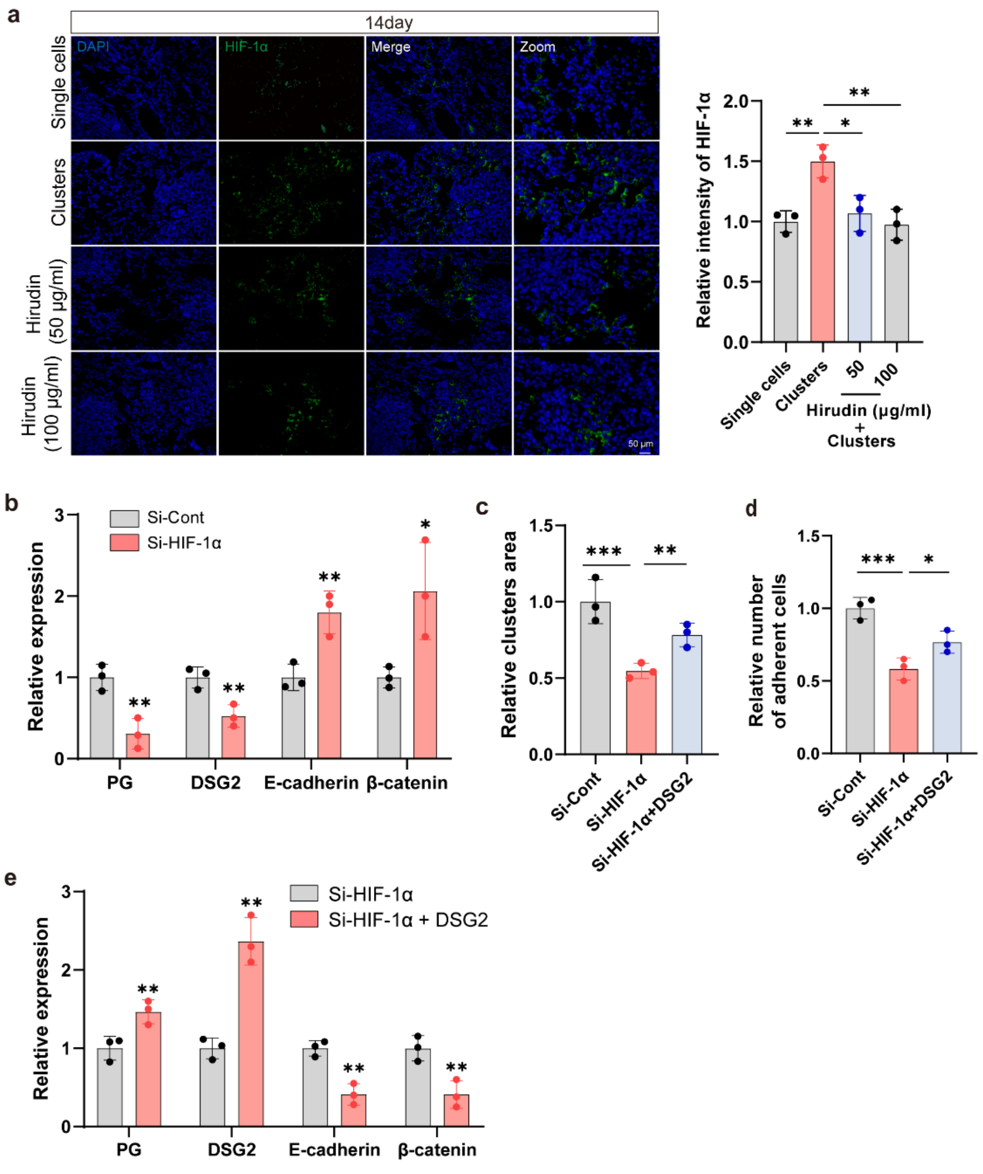

Supplementary Fig. 5. The role of HIF-1α in CTC cluster formation.

(a) Immunofluorescence images of HIF-1α in lung tissues 14 days after tail vein injection of 4T1 single cells, cell clusters, and hirudin-treated cell clusters, with quantification of positive areas. n= 3. (b) Relative protein expression of PG, DSG2, E-cadherin, and β-catenin after HIF-1α knockdown. (c-d) The relative cluster area and the number of adherent MCF-7-GFP cells of HIF-1α knockdown cell clusters treated with recombinant DSG2 protein. (e) The relative protein expression of PG, DSG2, E-cadherin, and β-catenin in HIF-1α knockdown MCF-7 cell clusters treated with or without recombinant DSG2 protein. n = 3. Data are mean±SD, **P<0.01, ***P<0.001.

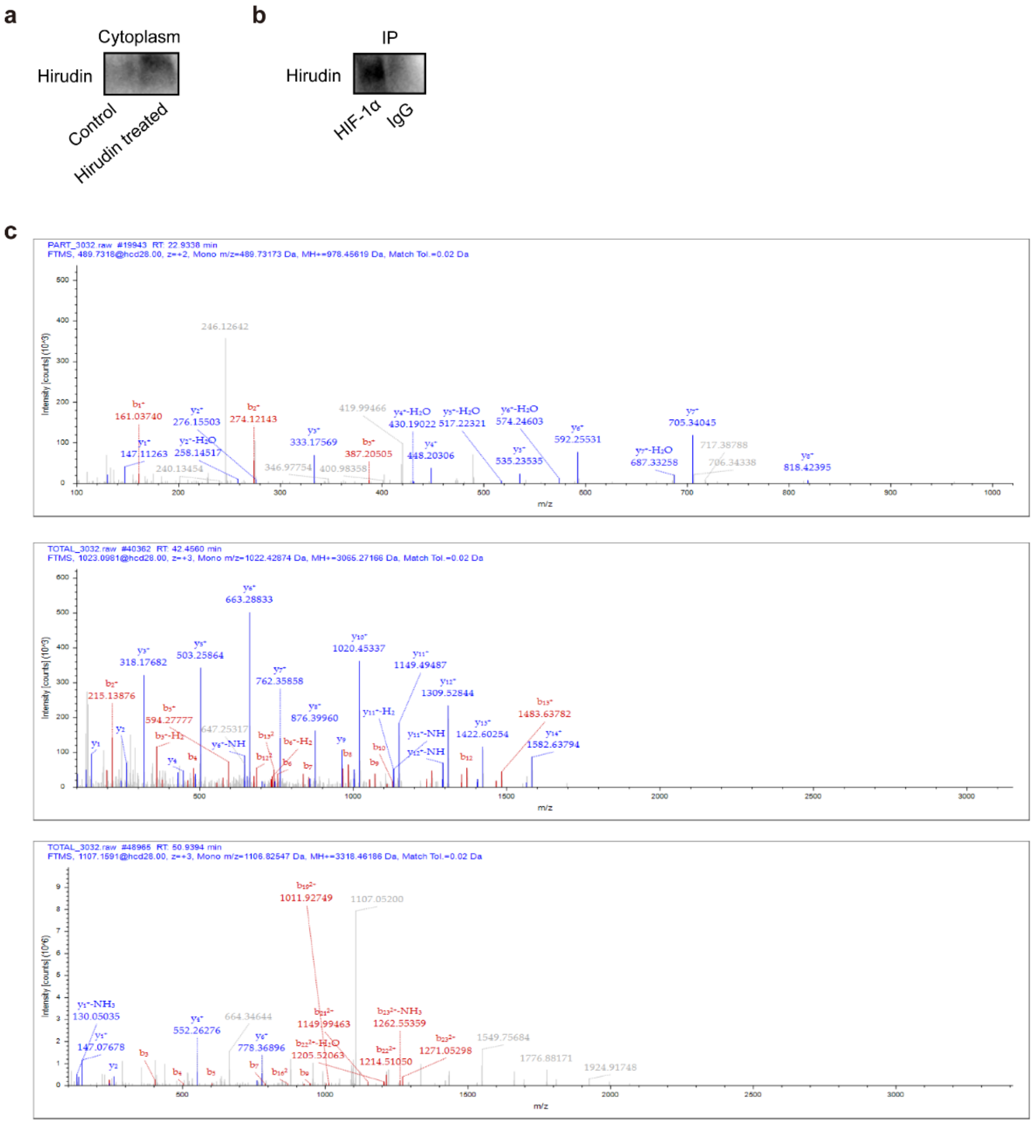

Supplementary Fig. 6. The detection of hirudin.

(a-b) MCF-7 cell clusters were treated with or without 100 μg/mL hirudin for 24 hours. The presence of hirudin in the (a) cytoplasm and (b) HIF-1α immunoprecipitates were detected by western blotting. (c) Mass spectra of the three unique peptides of hirudin in the HIF-1α immunoprecipitation sample.

Supplementary Tables

Supplementary Table 1. Key resources table

Reagent or Resource	Source	Identifier
Heparin	Wanbang Biopharmaceuticals	H32020612
Hirudin	Jin Yibai Biological Technology	000000153
Paraformaldehyde	Sigma-Aldrich	158127
Crystal violet	Sigma-Aldrich	C6158
Slide mounting medium	Sigma-Aldrich	06522
Opti-MEM medium	Gibico	31985070
Lipofectamine 3000	Invitrogen	L3000015
IHC Detection Kit	ZSGB-BIO	PV-9000
Diaminobenzidine	Beyotime	P0203
Hematoxylin	Beyotime	C0107
Triton X-100	Beyotime	ST795
DAPI	Beyotime	C1006
DPBS	Gibco	C14190500BT
Glutaraldehyde (2.5%)	LEAGENE	DF0156
RIPA Lysis Buffer	Beyotime	P0013B

BCA assay kit	Beyotime	P0012S
Ki67 Polyclonal antibody	Proteintech	28074-1-AP
DSG2 Polyclonal antibody	Proteintech	21880-1-AP
PG Polyclonal antibody	Proteintech	27872-1-AP
ZO-1 Polyclonal antibody	Proteintech	21773-1-AP
CEACAM6 Rabbit mAb	ABclonal	A5971
Vimentin mouse mAb	Santa Cruz Biotechnology	sc-6260
PD-L1/CD274 mAb	Proteintech	66248-1-lg
HIF-1α antibody	MCE	HY-P80704
E-cadherin antibody	Affinity Biosciences	AF0131
β-Catenin (D10A8) XP® Rabbit mAb	Cell Signaling Technology	8480S
Goat Anti-Mouse IgG H&L (FITC)	Abcam	ab6785
Goat Anti-Rabbit IgG H&L (TRITC)	Abcam	ab6718
GAPDH antibody	Bioworld	AP0063
Goat anti-rabbit IgG (H&L)-HRP	Bioworld	BS13278
Goat anti-mouse IgG (H&L)-HRP	Bioworld	BS12478
BeyoClick™ EdU Cell Proliferation Kit	Beyotime	C0075S
M-PER Mammalian Protein Extraction Reagent	Thermo	78501
Pronase	Roche	10165921001
EDTA	Macklin	C10097024

Supplementary Table 2. Information on hydrogen bonds formed between hirudin and HIF-1α at different time points

Time/ns	Hydrogen Bond	Distance/Å	H-Donor	H-Acceptor	∠C-H…O/°
0	α:Tyr276:HH - β:Asn20:OD1	2.82	α:Tyr276:HH	H:ASN20:OD1	133.58
	α:Asn326:HD22 - β:Gln24:OE1	1.93	α:Asn326:HD22	H:GLN24:OE1	161.45
	β:Val1:H1 - α:Thr296:OG1	2.76	β:Val1:H1	X:THR296:OG1	142.15
	β:VAl1:H3 - α:Phe295:O	2.47	β:VAl1:H3	X:PHE295:O	149.09
	β:Val21:H - α:Leu248:O	2.45	β:Val21:H	X:LEU248:O	113.87
	β:Gln24:HE21 - α:Gln331:O	2.51	β:Gln24:HE21	X:GLN331:O	114.82
	β:Gln24:HE22 - α:Pro332:O	1.82	β:Gln24:HE22	X:PRO332:O	163.03
30	α:Hid291:HD1 - β:Asn20:OD1	1.72	α:Hid291:HD1	β:Asn20:OD1	147.02
	α:Asn326:HD22 - β:Gln24:OE1	2.79	α:Asn326:HD22	β:Gln24:OE1	114.55
	α:Gln331:HE22 - β:Gln24:OE1	2.04	α:Gln331:HE22	β:Gln24:OE1	155.95
	β:Ser19:HG - α:Thr288:O	1.99	β:Ser19:HG	α:Thr288:O	130.45
	β:Asn20:HD21 - α:Tyr276:OH	2.98	β:Asn20:HD21	α:Tyr276:OH	97.03
	β:Asn20:HD22 - α:Leu248:O	2.12	β:Asn20:HD22	α:Leu248:O	162.21
	β:Gln24:H - α:Pro332:O	1.92	β:Gln24:H	α:Pro332:O	174.63

Supplementary Table 3. The detection of hirudin in the cytoplasm

Sample	Protein.Names	Stripped.Sequence	Precursor.Charge	Precursor.Id	H_cells
Hirudin treated group	Hirudin	CILGSDGEK	1	C(UniMod:4)ILGSDGEK1	7.26458e+07
		CILGSDGEK	2	C(UniMod:4)ILGSDGEK2	4.73041e+08
		CILGSDGEKNQCVTGEGTPK	3	C(UniMod:4)ILGSDGEKNQC(UniMod:4)VTGEGTPK3	1.9012e+06
		LTYTDCTESGQNLCLCEGSNVCGQGNK	3	LTYTDC(UniMod:4)TESGQNLC(UniMod:4)LC(UniMod:4)EGSNVC(UniMod:4)GQGNK3	1.52461e+08
		NQCVTGEGTPK	1	NQC(UniMod:4)VTGEGTPK1	1.07982e+06
		NQCVTGEGTPK	2	NQC(UniMod:4)VTGEGTPK2	1.10899e+07
		NQCVTGEGTPKPQSHNDGDFEEIPEEYLQ	3	NQC(UniMod:4)VTGEGTPKPQSHNDGDFEEIPEEYLQ3	2.74481e+08
		NQCVTGEGTPKPQSHNDGDFEEIPEEYLQ	4	NQC(UniMod:4)VTGEGTPKPQSHNDGDFEEIPEEYLQ4	1.15075e+07
		PQSHNDGDFEEIPEEYLQ	2	PQSHNDGDFEEIPEEYLQ2	9.80663e+06

The peptides of hirudin were detected.

Supplementary Table 4. HPLC-MS/MS analysis of hirudin peptides

Sample	Description	Coverage [%]	Peptides	Unique Peptides	AAs	MW [kDa]	calc. pI	Abundance
HIF-1α	Hirudin sequence	100	3	3	65	7	4.13	13283534.58±4330501.469
IgG	Hirudin sequence	14	1	1	65	7	4.13	–
